# The regulation of Hh/Gli1 signaling cascade involves Gsk3β- mediated mechanism in estrogen-derived endometrial hyperplasia

**DOI:** 10.1038/s41598-017-06370-1

**Published:** 2017-07-26

**Authors:** Jyoti Bala Kaushal, Pushplata Sankhwar, Suparna Kumari, Pooja Popli, Vinay Shukla, Mohd. Kamil Hussain, Kanchan Hajela, Anila Dwivedi

**Affiliations:** 1 Division of Endocrinology, CSIR-Central Drug Research Institute, Lucknow, 226031 U.P. India; 2 Department of Obstetrics & Gynecology, King George’s Medical University, Lucknow, 226001 U.P. India; 3 Division of Medicinal & Process Chemistry, CSIR-Central Drug Research Institute, Lucknow, 226031 U.P. India; 4 Academy of Scientific and Innovative Research (AcSIR), New Delhi, 110025 India

## Abstract

The present study was undertaken to explore the functional involvement of Hh signaling and its regulatory mechanism in endometrial hyperplasia. Differential expression of Hh signaling molecules i.e., Ihh, Shh, Gli1 or Gsk3β was observed in endometrial hyperplasial (EH) cells as compared to normal endometrial cells. Estradiol induced the expression of Hh signaling molecules and attenuated the expression of Gsk3β whereas anti-estrogen (K1) or progestin (MPA) suppressed these effects in EH cells. Cyclopamine treatment or Gli1 siRNA knockdown suppressed the growth of EH cells and reduced the expression of proliferative markers. Estradiol also induced the nuclear translocation of Gli1 which was suppressed by both MPA and K1 in EH cells. While exploring non-canonical mechanism, LY-294002 (Gsk3β activator) caused a decrease in Gli1 expression indicating the involvement of Gsk3β in Gli1 regulation. Further, Gsk3β silencing promoted the expression and nuclear translocation of Gli1 demonstrating that Gsk3β serves as a negative kinase regulator of Gli1 in EH cells. Similar attenuation of Hh signaling molecules was observed in rats with uterine hyperplasia undergoing anti-estrogen treatment. The study suggested that Hh/Gli1 cascade (canonical pathway) as well as Gsk3β-Gli1 crosstalk (non-canonical pathway) play crucial role in estrogen-dependent cell proliferation in endometrial hyperplasia.

## Introduction

Endometrial hyperplasia (EH) is a precancerous stage characterized by non-invasive proliferation of the endometrium^1, 2^. It is a pathological condition basically defined as proliferation of endometrial glands, or inner lining of the uterus and determined by hyper-estrogenism of exogenous or endogenous origin, with deficiency or absence of progesterone stimulus^3, 4^. Unopposed estrogen action causes excessive and abnormal proliferation of the glandular and stromal cells of the endometrium. These estrogen-induced changes in proliferation and morphogenesis culminate into the formation of atypical hyperplasia which subsequently leads to development of endometrial carcinoma^5, 6^. Although often asymptomatic, endometrial hyperplasia can present with abnormal uterine bleeding^7^.

The hedgehog (Hh) pathway is known as developmental signaling pathway involved in numerous fundamental processes in vertebrates embryonic developments including stem cell maintenance, determination of cell fate, tissue polarity, cell differentiation, and cell proliferation^8, 9^. Activated Hh signaling has been reported to play a potential role in development of the female reproductive tract by cell proliferation and differentiation in the neonatal uterus and vagina via regulating a range of signaling molecules^10^. In addition, a differential expression of Hh genes has also been observed in rat uterus during pregnancy^11^. Besides this, constitutive activation of Hh pathway has been identified in a variety of human malignancies and tumorigenesis including, pancreatic, skin, gastrointestinal, lung, cervical, prostate^12–18^ and hyperplasic condition in small subset of tissues as pituitary, cerebral and prostate^19–21^. Inappropriate or over-expression of Gli1^22^ (transcription factor, a key molecule of Hh signaling pathway) has been known to be involved in early events of endometrial tumorigenesis^23^. The extensive alterations in the expression pattern of Hh signaling molecules also suggest that Shh signaling network functions differently in normal and hyperplasic endometrium than under the carcinomatous condition^24^. However, steroid-regulatory mechanism and signaling cascade (ligand- dependent/canonical pathway and ligand- independent/non-canonical pathway) of the Hh signaling associated towards estrogen-mediated endometrial hyperplasia progression still remain unclear.

During canonical hedgehog signaling pathway, in the absence of ligand binding, the Hh receptor Patched (Ptch) blocks Smoothened (Smo) activity, which generates a truncated form of glioma-associated oncogene homolog proteins i.e. Gli, that ultimately represses a subset of Hh target genes. However, in the presence of ligand binding, Ptch receptor internalization occurs and hence, degradation of Hh-Ptch complex thereby stabilization of full length, transactivating Gli1 protein. The full length active form of Gli1 participates in regulating various cellular processes including cell proliferation and differentiation^25–28^. In non-canonical Hh signaling, the components signal outside the Hh-Ptch-Smo-Gli paradigm, that plays a crucial role in activation of molecular pathway by modulating activity of Gli1^29^, a key component of this signaling^22^.

In general, the molecular action of Gli is negatively regulated by multifunctional serine/threonine kinase glycogen synthase kinase-3β (Gsk3β)^30, 31^. Studies reported that Gsk3β act as bipotential in regulation of Gli1. It acts as a negative regulator (by phosphorylating Gli and promote its degradation) suppressing Hh signaling or as a positive regulator in stimulating Hh signaling^32, 33^. Interestingly, while exploring the role of Wnt/β-catenin pathway in regulation of estrogen action, it has been reported that decreased expression of Gsk3β via Lithium treatment encourages estradiol-induced proliferative and morphogenic changes in the uterus of mice leading to hyperplasia^34^. However, the effect of estrogen on Hh signaling molecules and Gsk3β/Gli1 cascade have not been studied in endometrial hyperplasia. We hypothesize that there might be a direct correlation of Gsk3β with Hh signaling in progression of estrogen- mediated cellular growth in endometrial hyperplasia. The current study was therefore, aimed to investigate the role of hedgehog signaling, and subsequently, the Gsk3β -mediated regulation of Gli1 in estrogen-dependent condition in endometrial hyperplasic cells. We investigated the role of Hh signaling (canonical and non-canonical pathway) in primary human endometrial hyperplasial cells and in rat uterine hyperplasia model under the influence of progestin (medroxyprogesterone acetate, MPA) and the potent anti-estrogenic agent (K1)^35, 36^ which shows antiproliferative potential in uterus^37, 38^. The study showed the crucial involvement of Hedgehog/Gli1 pathway and Gsk3β-mediated Gli1 crosstalk in estrogen-dependent endometrial hyperplasial cell proliferation.

## Results

### Hedgehog signaling molecules i.e., Ihh, Shh, Gli1 or Gsk3β are differentially expressed in normal and hyperplasial cells of human endometrium

**Figure 1:**
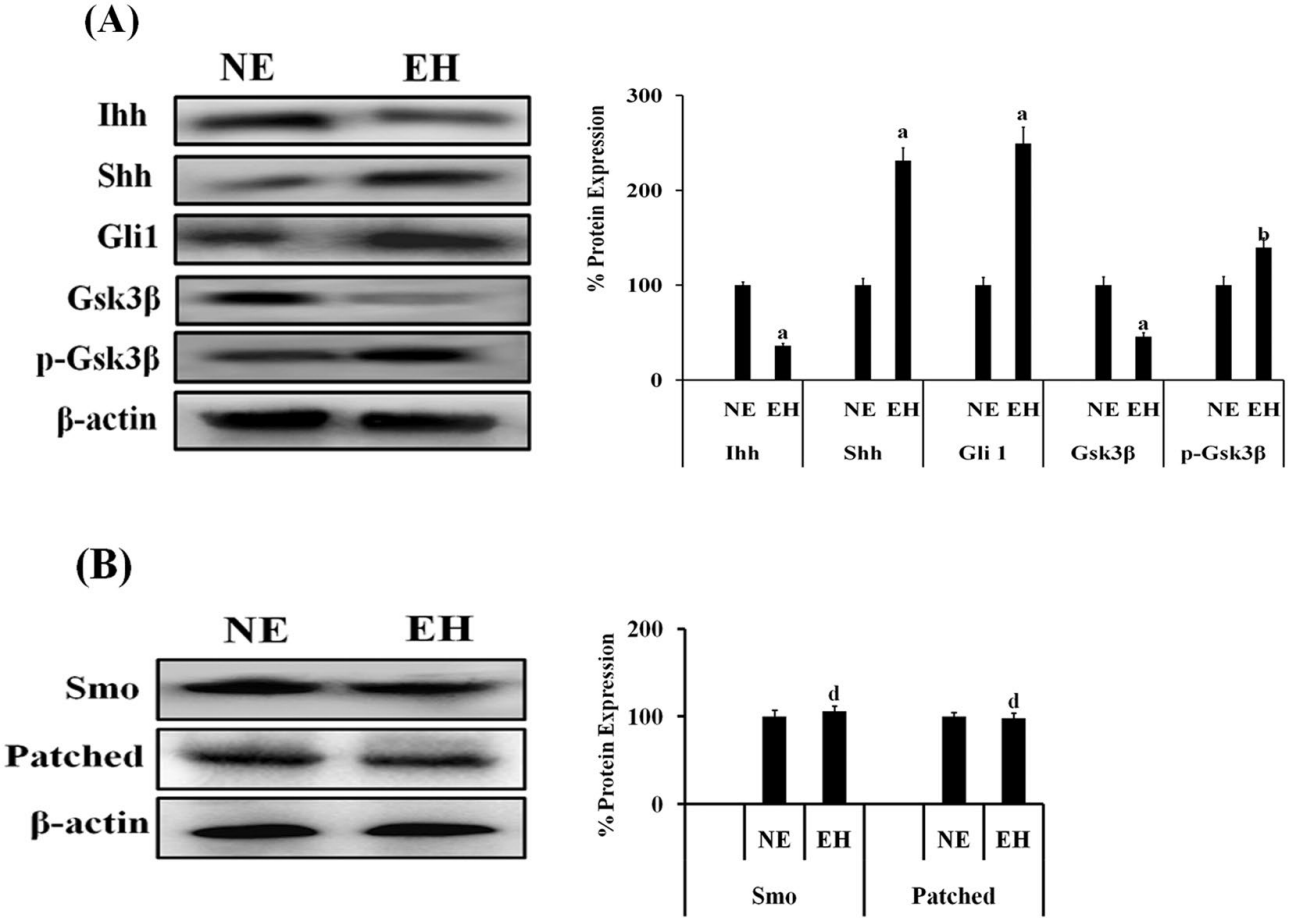
Differential expression of Hh signaling molecules in primary human normal endometrial (NE) and primary human endometrial hyperplasial (EH) cells. Representative western blots showing the expression pattern of Hh signal-related molecule like Ihh, Shh, Gli1, Gsk3β and p- Gsk3β (**A**); Patched, Smo (**B**) in primary human NE and EH cells. Cells were maintained in MEM media with 10% FBS. Whole cell lysates (20 μg) were subjected to SDS-PAGE and western blot analysis. β-actin was used as internal loading control. Densitometric data shown as % change in protein expression levels. Values are expressed as mean ± SEM (n = 3 independent samples), p values: ^a^p < 0.001, ^b^p < 0.01, ^c^p < 0.05 and ^d^p > 0.05 vs. control.

The analysis of Hh signaling molecules involved in canonical Hh signaling such as Ihh, Shh, Gli1, Patched and Smo and Gsk3β, p-Gsk3β involved in non-canonical Hh pathway, in human endometrial hyperplasial (EH) cells as compared to normal endometrial cells (NE) was done by western blotting. A significant reduction was observed in Ihh and Gsk3β protein expression, whereas induction was found in the protein expression of Shh, Gli1 and p-Gsk3β in EH as compared to NE cells (Fig. 1A). Simultaneously, we did not find any changes in protein expression level of Smo and Patched (Fig. 1B). The densitometric analysis revealed that the expression of Ihh and Gsk3β was reduced by ~65% (p < 0.001) and ~50% (p < 0.001) respectively whereas the expression of Shh, Gli1and p- Gsk3β was increased by ~130% (p < 0.001), ~145% (p < 0.001), and ~40% (p < 0.01) respectively, in EH cells as compared to NE cells.

### Endometrial hyperplasial cell viability is inhibited by the anti-estrogenic agent (K1)

The effect of anti-estrogenic agent (K1) on cell viability was examined by MTT assay. Anti-estrogen agent (K1) reduced the viability of human endometrial hyperplasia cells in a dose-dependent manner with IC^50^ of 5 μM (p < 0.001). However, K1 was ineffective in normal endometrial primary culture cells. Results showed that K1 has an anti-proliferative effect on primary EH cells without affecting the NE cells (Supplementary Fig. 1A). Hence, to delineate the estrogen-mediated regulatory mechanism of Hh signaling, K1 was used in subsequent experiments.

### Anti-estrogenic agent (K1) or progestin (MPA) alters the expression profile of hedgehog signaling molecules and proliferative markers in primary human endometrial hyperplasial cells

**Figure 2:**
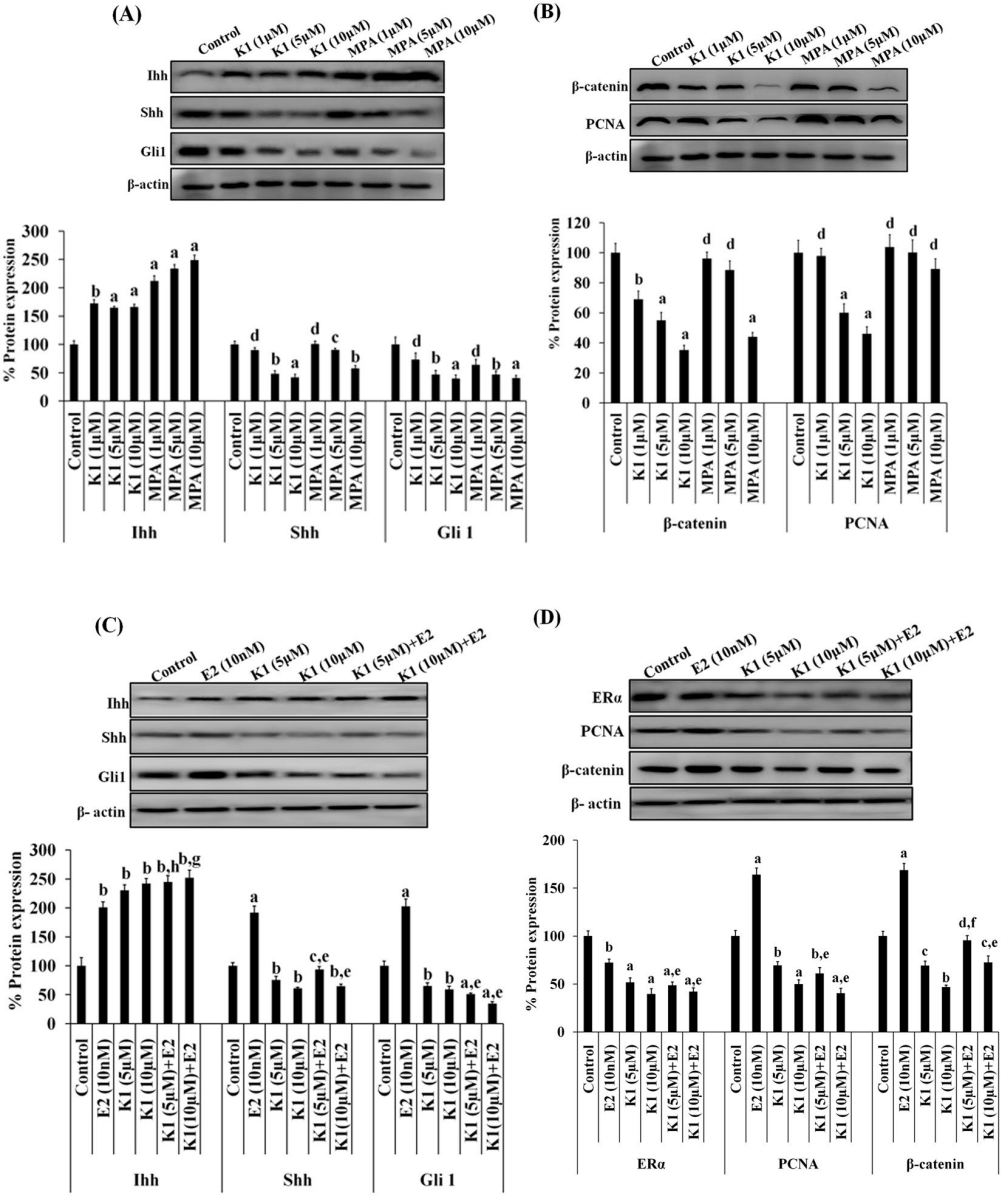
Anti-estrogenic agent (K1) or progestin (MPA) antagonizes estrogen- induced activation of Hh signaling molecules and proliferative markers in primary human EH cells. Representative western blots showing expression profile of Hh signaling molecules such as Ihh, Shh and Gli1 (**A**), and proliferative markers such as PCNA and β-catenin (**B**) in primary human EH cells treated with different concentrations of K1 or MPA. Representative western blots showing expression profile of Hh signaling molecules such as Ihh, Shh, Gli1 (**C**), and proliferative markers such as ERα, PCNA and β-catenin (**D**) in primary human EH cells treated with vehicle, E2 or anti-estrogenic agent K1 (5 μM or10 μM) with absence and presence of E2 (10 nM) for 48 h. β-actin was used as internal loading control. Densitometric data shown as % change in protein expression levels. Values are expressed as mean ± SEM, (n = 3 independent samples). p values: ^a^p < 0.001, ^b^p < 0.01, ^c^p < 0.05 and ^d^p > 0.05 vs. control; ^e^p < 0.001, ^f^p < 0.01, ^g^p < 0.05 and ^h^p > 0.05 vs. E2 - treated group.

The effect of anti-estrogenic agent (K1) or progestin (MPA) or β-estradiol (E2) on protein expression profile of Hh signaling molecules and proliferation markers in primary EH cells was analyzed (Fig. 2). A significant reduction in Shh, Gli1, PCNA, β-catenin expression was observed in K1 and MPA treated groups in dose-dependent manner (Fig. 2A and B). Interestingly, Ihh expression was found to be increased in all K1 and MPA treated groups (Fig. 2A). Densitometric analysis revealed that K1 (5 μM) reduced the expression of Shh by~45% (P < 0.01), Gli1 by ~55% (p < 0.01), β-catenin by ~50% (p < 0.001) and PCNA by ~45% (p < 0.01) whereas the expression of Ihh by ~95% (p < 0.001) was found to be increased, as compared to control untreated EH cells (Fig. 2A and B). MPA (5 μM) reduced the expression of Shh by ~30% (p < 0.05), Gli1 by ~60% (p < 0.01) while induced the expression of Ihh by ~140% (p < 0.001) as compared to control untreated EH cells (Fig. 2A). Apart from this, β-estradiol up-regulated the expression of Ihh, Shh, Gli1, PCNA and β-catenin (p < 0.001), whereas down-regulated the expression of ERα by ~45% (p < 0.01) as compared to control (Fig. 2C and D). While this β-estradiol induced effect was found to be abolished when EH cells were treated with K1 in presence of E2. Results showed a significant decrease in the expression of Shh, Gli1, ERα, PCNA (p < 0.001) and β-catenin (p < 0.01) whereas the expression of Ihh was increased (p < 0.05) in K1 + E2 treated cells as compared to E2-treated EH cells (Fig. 2C and D). These data indicated that K1 and MPA exert significant effect on Hh signaling in EH cells. Induction of expression of Hh signaling molecules and proliferative markers by β-estradiol and reversal of these estrogenic effects by K1 and MPA suggested the involvement of Hh signaling in estrogen- mediated cellular proliferation of EH cells.

### Estrogen up-regulated the expression of Gli1 and its nuclear translocation while K1 or MPA counteracted its expression in endometrial hyperplasial cells

**Figure 3:**
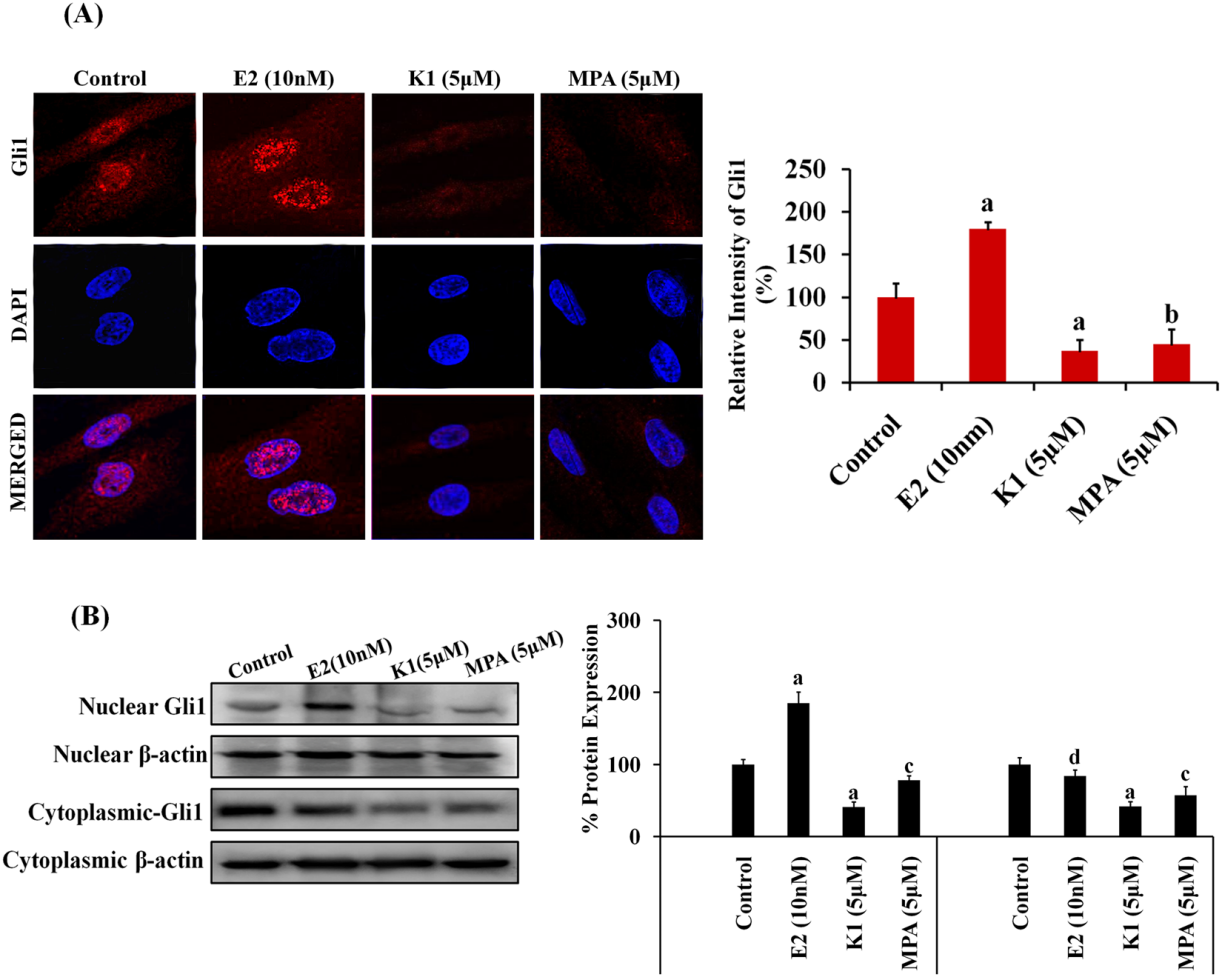
Estrogen up-regulates the expression of Gli1 and its nuclear localization while K1 or MPA counteracts its expression in primary human endometrial hyperplasial cells. (**A**) Representative micrographs (Left panel) demonstrating the nuclear localization of Gli1 via confocal microscopy to detect the effect of E2 or K1 or MPA in primary human EH cells. EH cells were treated with vehicle or E2 (10 nM) or K1 (5 µM) or MPA (5 µM) for 24 h. Cells were fixed, permeabilized, incubated with Gli1 antibody as described in ‘materials and methods’ section. Experiments were repeated at least three times (independent samples). The fluorescence intensity was determined by LSM Image Browser software (Right panel). Values are expressed as mean ± SEM, n = 3 (independent samples). p values: ^a^p < 0.001, ^b^p < 0.01 vs. control. (**B**) Representative western blots showing the nuclear and cytosolic Gli1 expression in EH cells. Nuclear and cytosolic proteins were extracted following manufacturer’s instructions and subjected to western blotting using anti-Gli1 antibody. β-actin was used as internal loading control. Densitometric data shown as % change in protein expression levels. Values are expressed as mean ± SEM, n = 3 (independent samples). p values: ^a^p < 0.001, ^c^p < 0.05 and ^d^p > 0.05 vs. control.

It has been known earlier that Gli1 (transcription factor) acts as a key molecule for Hh pathway activation^22^. Thus, to explore the estrogen-induced activation of Hh signaling pathway, the nuclear translocation of Gli1 in primary EH cells was assessed (Fig. 3). For confocal microscopic experiment, primary EH cells were treated with β-estradiol (10 nM), K1 (5 μM) and MPA (5 μM) for 24 h of duration. Fluorescence intensity analysis of images revealed that β-estradiol significantly induced the expression of Gli1 (p < 0.001) and promotes its nuclear localization while both K1 and MPA reduced its expression in both cytosolic and nuclear compartment (Fig. 3A). Simultaneously, these results were confirmed by western blot analysis to see the nuclear and cytosolic protein expression of Gli1 protein in EH cells. A decline in expression level of Gli1 (p < 0.001) in subcellular region in K1 or MPA treated EH cells was observed whereas β-estradiol- treated cells showed increased nuclear expression of Gli1 (~70%, p < 0.001) (Fig. 3B). Hence, these experimental results indicate that estrogen exposure enhanced the nuclear translocation of Gli1 as contrast to that caused by K1 and MPA diminished estrogenic effect, further corroborating regulation of Gli1 expression by estrogen in EH cells.

### Effect of cyclopamine, hedgehog signaling inhibitor or knockdown of Gli1 caused growth inhibition of endometrial hyperplasial cells

**Figure 4:**
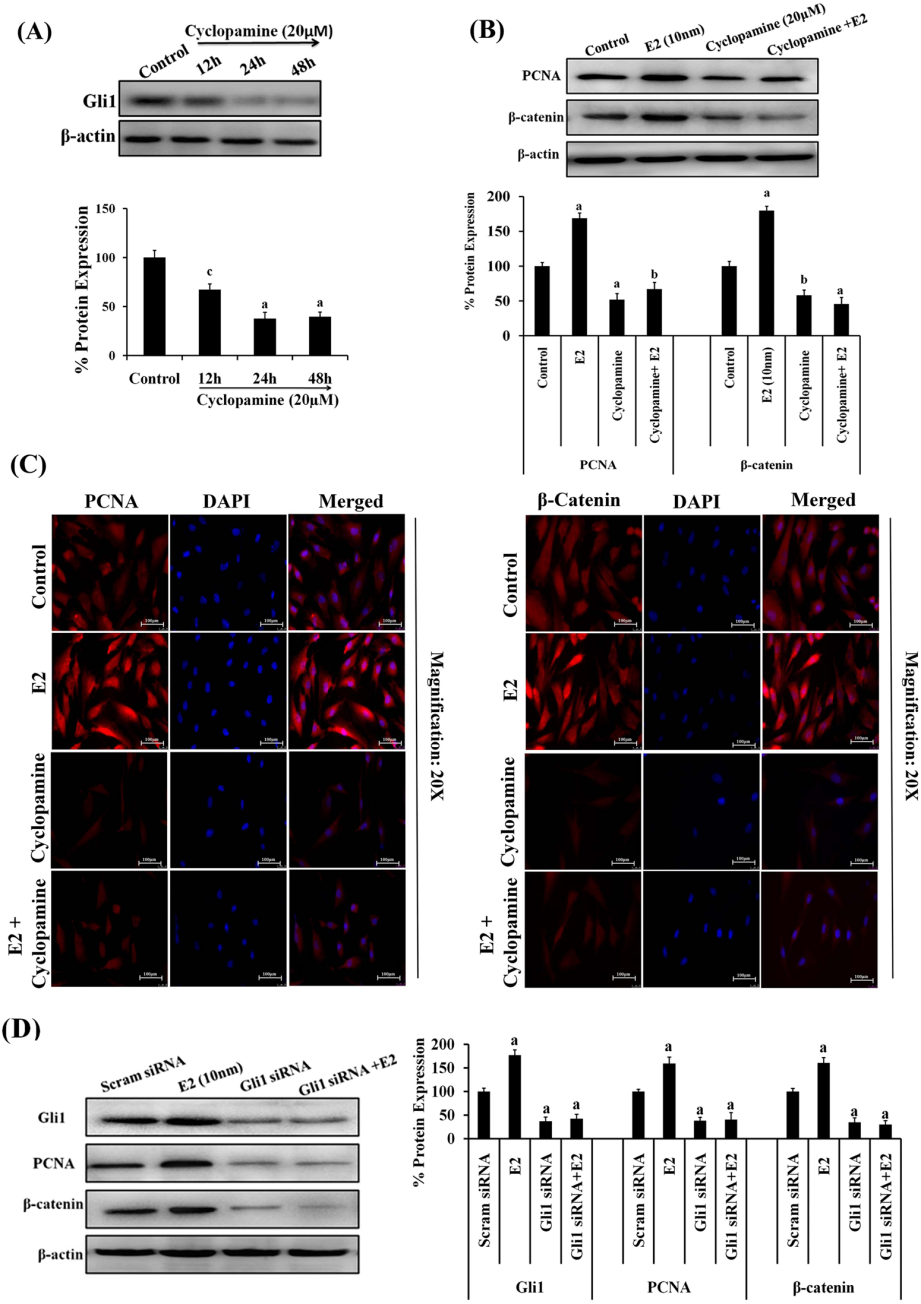
Effect of cyclopamine, Hh signaling inhibitor or knockdown of Gli1 on growth inhibition of endometrial hyperplasial cells. (**A**) Representative western blots showing a decline in Gli1 expression in time- dependent manner (12 h, 24 h, 48 h) in EH cells treated with cyclopamine. β-actin was used as internal loading control. Densitometric data shown as % change in protein expression levels. Values are expressed as mean ± SEM, n = 3 (independent samples). p values: ^a^p < 0.001, ^c^p < 0.05 vs control. (**B**) Representative western blot images illustrating the effect of cyclopamine on proliferative markers (PCNA and β-catenin) in primary EH cells. EH cells were treated with vehicle or E2 or cyclopamine alone or alongwith E2 for 24 h. Densitometric data shown as % change in protein expression levels. Values are expressed as mean ± SEM, n = 3 (independent samples). p values are: ^a^p < 0.001, ^b^p < 0.01, vs. control. (**C**) Immunofluorescence images demonstrating the distribution of PCNA or β-catenin in EH cells pre-treated with cyclopamine in presence and absence of estrogen for 24 h. Experiments were repeated at least three times. (**D**) Representative western blots images depicting the effect of Gli1 silencing on expression of proliferative markers as PCNA or β-catenin. β-actin was used as internal loading control. Densitometric data shown as % change in protein expression levels. Values are expressed as mean ± SEM, n = 3 (independent samples). p values: ^a^p < 0.001 vs. control.

To analyze the involvement of Hh signaling in cellular proliferation of EH cells, inhibition of Hh signaling was attempted by subjecting the cells to treatment of cyclopamine, a selective inhibitor of the Hh signaling pathway and the effect on proliferation was determined by MTT assay. In EH cells, cyclopamine- induced growth suppression was observed as early as 48 h after the addition of cyclopamine in a dose-dependent manner (Supplementary Fig. 2A). At 48 h, cyclopamine induced the significant growth suppression at 20 μM concentration (p < 0.001) (Supplementary Fig. 2A). Additionally, cyclopamine-treated cells showed suppression in cellular proliferation in presence of E2 (Supplementary Fig. 2D). We also demonstrated the effect of cyclopamine on inhibition of Hh signaling by determining Gli1 expression (12 h, 24 h, 48 h) in EH cells (Fig. 4A). A decline in expression of Gli1 was detected at 12 h (p < 0.05) which was more significant (p < 0.001) at 24 h and 48 h. In order to check if the loss in cell viability was due to induction of apoptosis, we analyzed Annexin V/PI stained cells by flow cytometry. We observed increased percentage of apoptotic cells in cyclopamine (20 µM) treated group at 24 h. The apoptotic cell fraction was approximately 40% higher as compared to control EH cells (Supplementary Fig. 2C).

Western blotting and immunocytochemistry was performed to detect the effect of cyclopamine on proliferative markers (Fig. 4B and C). A significant reduction in expression of PCNA and β-catenin (by ~50%) was observed in EH cells treated with cyclopamine alone or in combination of E2 (Fig. 4B). Immunofluorescence images also showed reduction in expression of PCNA and β-catenin in EH cells in presence of cyclopamine (alone or along with E2) (Fig. 4C).

As the effect of inhibition of Hh signaling was prominent on cellular proliferation of EH cells, we further examined the involvement of Gli1 in estrogen- mediated cellular proliferation by knock down of Gli1 by using siRNA. We found approximately ~75% (p < 0.001) knock down of Gli1 protein levels as compared to scrambled or control EH cells as determined by western blotting (Supplementary Fig. 3B). Further, the silencing of Gli1 significantly attenuated the expression of PCNA and β-catenin by ~55–65% (p < 0.001) in both groups (Fig. 4D). These findings clearly indicate that Gli1, a key regulatory protein of hedgehog pathway might play crucial role in estrogen -mediated proliferation of endometrial hyperplasia.

### Estrogen diminished the expression of Gsk3β in endometrial hyperplasial cells while K1 or MPA enhanced its expression

**Figure 5:**
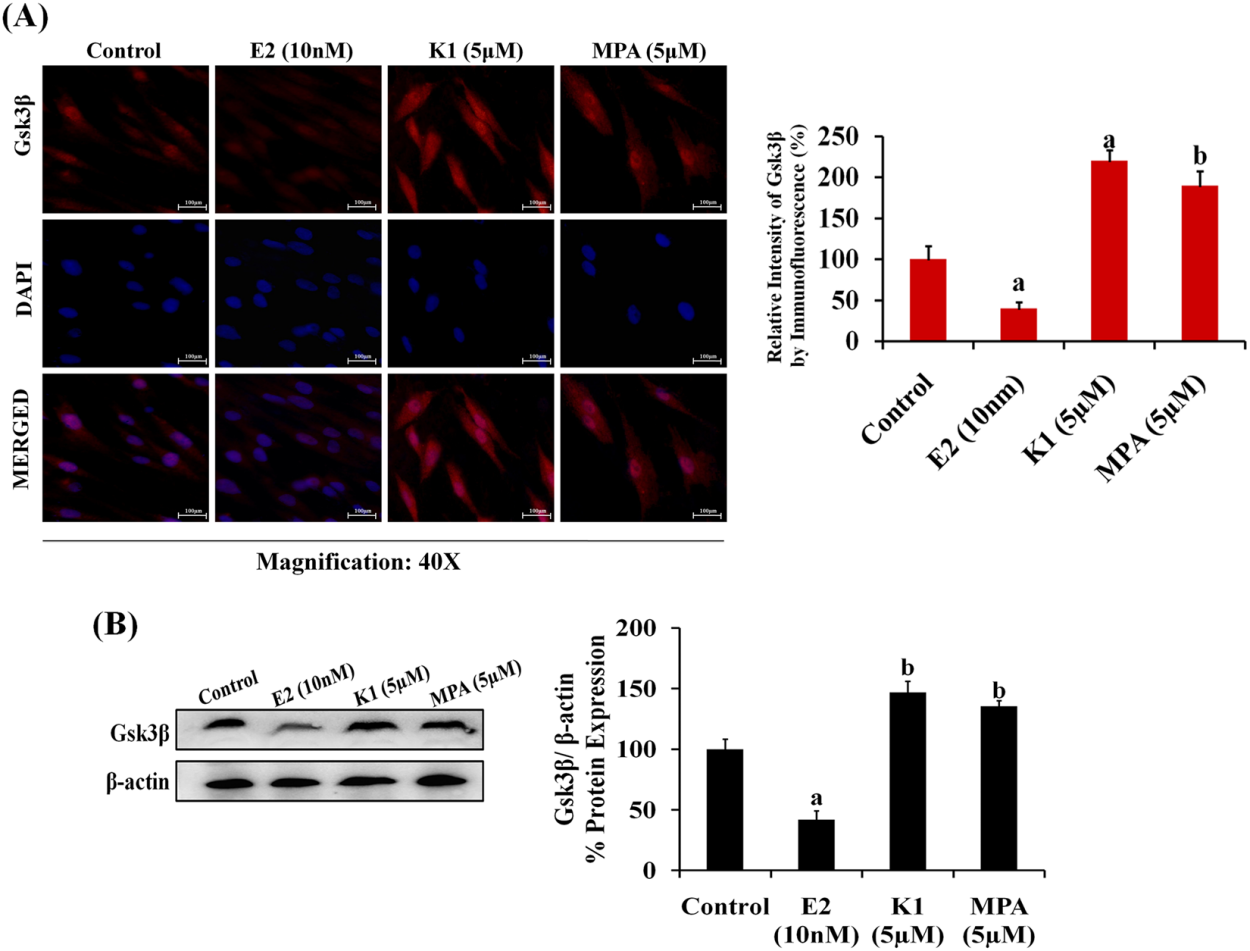
Estrogen diminishes the expression of Gsk3β in endometrial hyperplasial cells while K1 or MPA stimulates its expression. (**A**) Immunofluorescence images showing the effect of E2, K1 or MPA on sub-cellular expression of Gsk3β protein in EH cells (Left panel). Cells were treated with vehicle or E2 (10 nM) or K1 (5 µM) or MPA (5 µM) for 24 h. Cells were fixed, permeabilized, incubated with Gsk3β antibody for overnight as depicted in ‘materials and methods’ section. The Immunofluorescence intensity was quantified by NIS Elements software (Right panel). Values are expressed as mean ± SEM, n = 3 (independent samples). p values: ^a^p < 0.001, ^b^p < 0.01 vs. control. (**B**) Representative western blot images illustrating the expression of Gsk3β in primary EH cells –treated with E2, K1 and MPA. Membranes were stripped and re-probed with β-actin used as a control to correct for loading. Densitometric data shown as % change in protein expression levels. Values are expressed as mean ± SEM, n = 3 (independent samples). p values: ^a^p < 0.001, ^b^p < 0.01 vs. control.

Considering the possible role of Gsk3β in estrogen-dependent endometrial hyperplasia, we detected the expression profile of Gsk3β in presence of E2 or K1 or MPA, by immunofluorescence. Results revealed that E2 significantly decreased the Gsk3β expression (p < 0.001) while K1 (p < 0.001) or MPA (p < 0.01) induced its expression (Fig. 5A). In western blotting experiment, E2 was found to significantly down-regulate the expression of Gsk3β (p < 0.001) while K1 and MPA up-regulated its expression. (p < 0.01) (Fig. 5B). Results clearly indicated that estrogen exposure attenuates the Gsk3β expression in EH cells. Thus, failure of Gsk3β-mediated regulation might be one of the possible reasons for the constitutive activation of hedgehog signaling leading to continuous proliferation in EH cells.

### Gsk3β acted as a negative kinase regulator of hedgehog pathway that suppresses the activity of Gli1 in primary human endometrial hyperplasial cells

**Figure 6:**
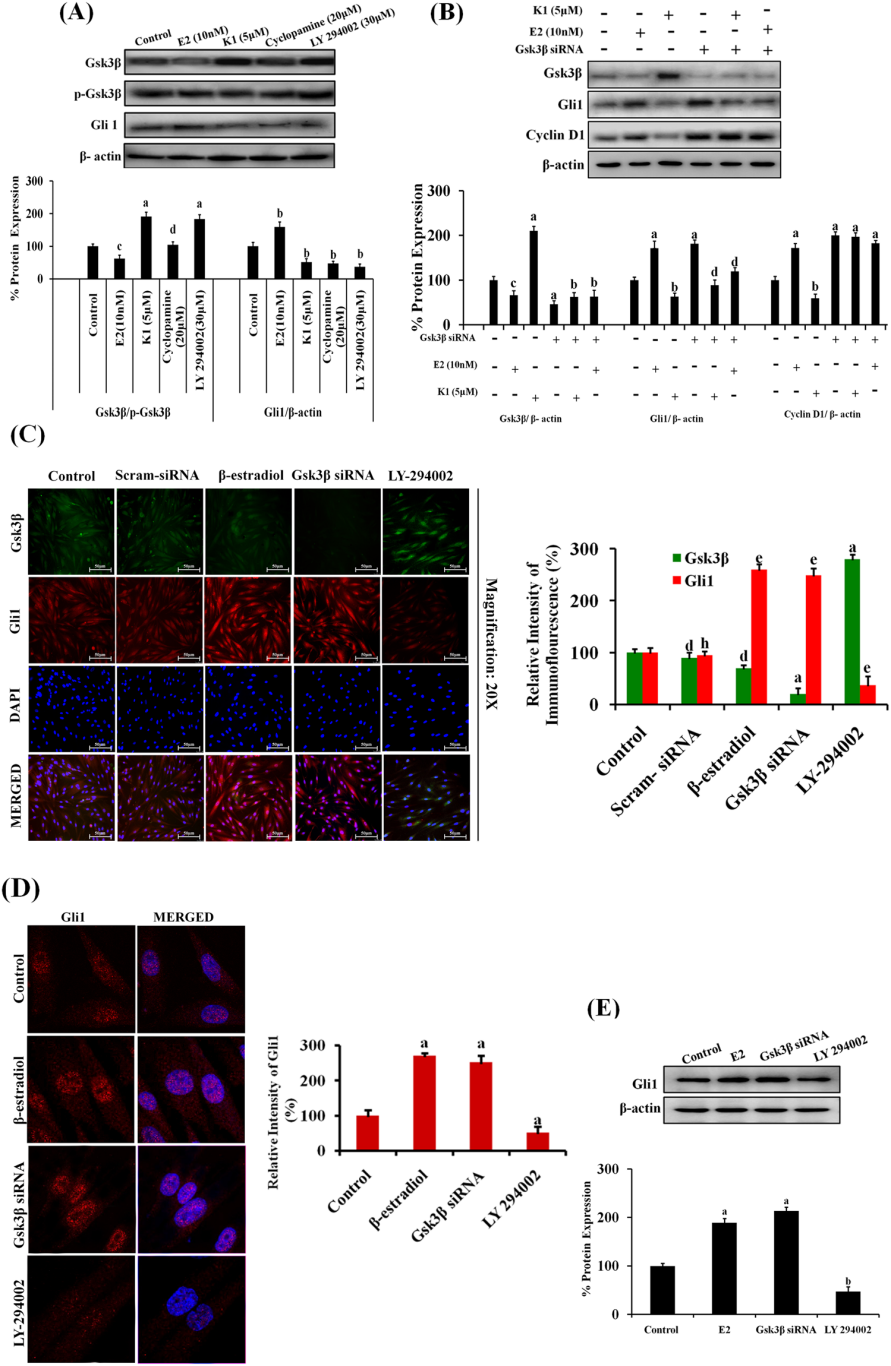
Gsk3β acts as a negative kinase regulator of Hh pathway that suppresses the activity of Gli 1 in primary human endometrial hyperplasial cells. (**A**) Representative western blot images showing the effect of Gsk3β activation on expression of Gli1 in primary EH cells. For this, EH cells were treated with E2 (10 nM) or K1(5 µM) or LY- 294002 (30 µM) or cyclopamine (20 µM) for 24 h. β-actin was used as internal loading control. Densitometric data shown as % change in protein expression levels. Values are expressed as mean ± SEM, n = 3 (independent samples). p values are: ^a^p < 0.001, ^b^p < 0.01, ^c^p < 0.05 and ^d^p > 0.05 vs. control. (**B**) siRNA transfection assay to demonstrate whether E2 induced activation of Gli 1 expression occurs via inhibition of Gsk3β. Primary human EH cells were transfected with 50 pmols of Gsk3β siRNA and incubated for 6 h at 37 °C followed by incubation with K1 or E2 for 48 h. β-actin was used as internal loading control. Densitometric data shown as % change in protein expression levels. Values are expressed as mean ± SEM, n = 3(independent samples). p values are: ^a^p < 0.001, ^b^p < 0.01, ^c^p < 0.05 and ^d^p > 0.05 vs. control. (**C**) Imunofluorescence images demonstrating the co-expression of Gsk3β and Gli1 in EH cells (Left panel). Cells were transfected with scramble or Gsk3β siRNA or treated with LY-294002 or E2. Cells were fixed, permeabilized, incubated with Gsk3β and Gli1 antibody for overnight as described in ‘materials and methods’ section. The Immunofluorescence intensity was quantified by Leica LAS Image analysis software (Right panel). Values are expressed as mean ± SEM, n = 3 (independent samples). p values: ^a^p < 0.001, ^b^p < 0.01, ^c^p < 0.05 and ^d^p > 0.05 vs. control for Gsk3β expression; ^e^p < 0.001, ^f^p < 0.01, ^g^p < 0.05 and^ h^p > 0.05 vs. control for Gli1 expression. (**D**) Confocal microscopy micrographs showing nuclear translocation of Gli1 protein in Gsk3β transfected- or LY-294002- or E2- treated EH cells. Images were grasped at 63X using Carl Zeiss LSM 510 META microscope (Left panel).The fluorescence intensity was determined by LSM Image Browser software (Right panel). Values are expressed as mean ± SEM, n = 3 (independent samples). p values: ^a^p < 0.001 vs. control. (**E**) Representative western blot images showing the expression of Gli1 in E2- treated, Gsk3β transfected- and LY-294002- treated EH cells. β-actin was used as internal loading control. Densitometric data shown as % change in protein expression levels. Values are expressed as mean ± SEM, n = 3 (independent samples). p values: ^a^p < 0.001, ^b^p < 0.01 vs. control.

In order to check the regulatory crosstalk mechanism between Gsk3β and Gli1, we treated EH cells with β-estradiol, anti-estrogenic agent (K1), a selective Gsk3β activator LY-294002^39^ and the cyclopamine for 48 h (Fig. 6A). LY294002- treated cells showed significantly decreased expression of Gli1 (p < 0.01) and the increased expression of Gsk3β (p < 0.001) while cyclopamine- treated cells showed reduced expression of Gli1 without any effect on Gsk3β expression, as compared to control EH cells (Fig. 6A). However, no difference in protein expression level of p-Gsk3β was observed in these cells. These data implicated that activation of Gsk3β by LY-294002, induced inhibition of Gli1 similar to that observed in cyclopamine- treated cells (Fig. 6A). Interestingly, K1 showed evidence of involvement of Gsk3β mediated regulation of Gli1 pathway by inducing expression of Gsk3β by ~95% (p < 0.001) and inhibiting Gli1 by ~50% (p < 0.01) expression (Fig. 6A). These data suggested that Gli1 is downstream target of Gsk3β in EH cells.

Concomitantly, we validated Gsk3β/Gli1 regulatory mechanism by silencing of Gsk3β via siRNA transfection. The transfection efficiency was determined by western blotting in hyperplasial cells transfected with scrambled or Gsk3β siRNA after 24 h of transfection. We observed approximately ~70% (p < 0.001) knock down of Gsk3β protein level as compared to scrambled treated groups (Supplementary Fig. 3A). In addition, immunoblotting analysis revealed that Gsk3β silencing caused upregulation of Gli1 by ~80%, and of cyclin D1 (a well known target of Gsk3β) expression by ~75% in siRNA- transfected cells. We found that E2 reduced the expression of Gsk3β along with increased expression of Gli1. However, in Gsk3β siRNA -transfected cells, E2 was not able to increase the Gli1 expression (Fig. 6B). Also, K1 increased the Gsk3β expression with simultaneous decrease in Gli 1 and cyclin D1 expression in EH cells. Interestingly, K1 did not show any effect on Gli1 expression in Gsk3β siRNA- transfected cells (Fig. 6B). These results showed that regulation of Gli1 expression is Gsk3β-mediated and the estrogen- induced Gli1 expression is subjected to Gsk3β regulation in EH cells (Fig. 6B).

We, further investigated the potential role of Gsk3β in Gli1 regulation by studying co-expression of Gsk3β and Gli1 protein and nuclear translocation of Gli1, in Gsk3β siRNA- transected and LY-294002- treated EH cells (Fig. 6C and D). The image analysis revealed that silencing of Gsk3β significantly reduced the expression of Gsk3β (p < 0.001), and induced the expression of Gli1 (p < 0.001) as compared to that in scrambled siRNA- transfected cells. LY-294002 -treated cells exhibited increased expression of Gsk3β along with reduced expression of Gli1 (p < 0.001) (Fig. 6C). These observations suggested that declined Gli1 levels were accompanied by an accumulation of Gsk3β or vice-versa. The study showed that Gsk3β knock down encouraged the nuclear translocation of Gli1 (Fig. 6D). On the other hand, the activation of Gsk3β by LY-294002 treatment mitigated the Gli1 expression in cytoplasmic and nuclear compartments, as compared to control (vehicle treated) EH cells (Fig. 6D). Similar results were obtained by western blot analysis (Fig. 6E). These results provided evidence for the involvement of Gsk3β- mediated transactivation of Gli1 in estrogen- induced hyperplasic condition.

### Effect of K1 or MPA treatment on uterine mass growth and expression of proliferative markers in uterus of rat with experimentally induced hyperplasia

Followed by *in vitro* experiments, we performed *in vivo* experiment in rat hyperplasial model under the influence of K1 or MPA for exploration of significant involvement of Hh signaling in endometrial hyperplasia. We observed a significant increase in wet uterine mass of ovariectomized rats receiving β-estradiol, as compared to that of control (vehicle administered) group (Supplementary Fig. 4A). A significant decrease in uterine weight (p < 0.001) was observed in rats receiving different doses of K1 or MPA along with β- estradiol, as compared to β-estradiol -treated group (36d) (Supplementary Fig. 4A).

The uterine histomorphometric analysis also showed extensive proliferation of endometrial epithelium in β-estradiol-treated rats (Supplementary Fig. 4B). In β-estradiol- administered rats (36 d), uterine sections showed an increase in endometrial area by ~270%, in luminal area by ~180%, in luminal epithelial cell height by ~160%, in glandular area by ~120%, in stromal area ~285%, and by ~80% in the ratio of glandular vs stromal area, as compared to that of control group. All these changes were found to be statistically significant (p < 0.001) and were indicative of the development of uterine hyperplasic conditions. A decrease in each of these parameters were observed in dose-dependent manner (100, 200, 400 µg/kg) when K1 alongwith β-estradiol was given to rats as compared to β-estradiol- treated group. MPA administered rats (25 mg/kg) also showed decrease in all histomorphometric parameters but the effect was highly significant (p < 0.001) in K1 administered rats at 400 µg/kg dose (Supplementary Fig. 4B).

**Figure 7:**
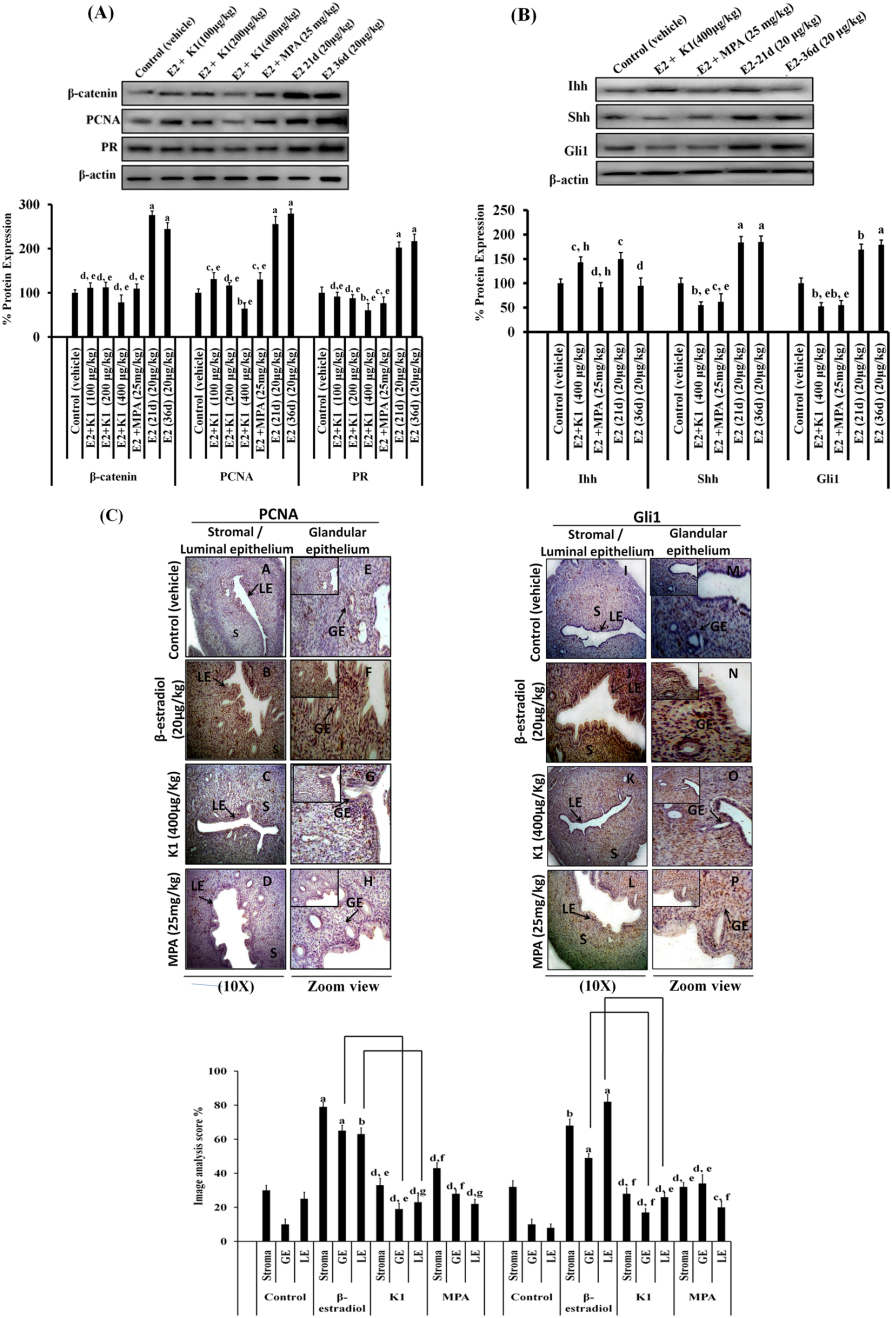
Anti-estrogenic agent (K1) or progestin (MPA) treatment antagonized estrogen- induced activation of Hh signaling molecules in rat uterine tissue. (**A**) Representative western blot images showing the expression of proliferative markers such as PR, PCNA and β- catenin in uterine tissue of rats treated with β- estradiol (E2), K1 or MPA. β-actin was used as internal loading control. Densitometric data shown as % change in protein expression levels. Results are expressed as mean ± SEM, n = 3 (independent samples). p values: ^a^p < 0.001, ^b^p < 0.01, ^c^p < 0.05 and ^d^p > 0.05 vs. control; ^e^p < 0.001, ^f^p < 0.01, ^g^p < 0.05 and ^h^p > 0.05 vs. E2 (36d) administered groups. (**B**) Representative western blots illustrating the expression of Ihh, Shh and Gli1 in rat uterine tissue. Densitometric data shown as % change in protein expression levels. Values are expressed as mean ± SEM, n = 3 (independent samples). p values are: ^a^p < 0.001, ^b^p < 0.01, ^c^p < 0.05 and ^d^p > 0.05 vs. control; ^e^p < 0.001, ^f^p < 0.01, ^g^p < 0.05 and ^h^p > 0.05 vs. E2 administered group. (**C**) Left panel (showing immunohistochemical localization of PCNA): Control (A- S/LE, E- GE), E2 (B- S/LE, F- GE), K1 (C- S/LE, G- GE), MPA (D- S/LE, H- GE); Right panel (showing immunohistochemical localization of Gli1): Control (I- S/LE, M- GE), E2 (J- S/LE, N- GE), K1 (K- S/LE, O- GE), MPA (L- S/LE, P- GE). Image analysis of PCNA or Gli1 in rat uterine tissue and staining intensity of all these proteins were quantified by image analysis software ‘Image-Pro Plus 4.0’ (Maryland, USA). Values are expressed as mean ± SEM, n = 3 in all the groups. p values: ^a^p < 0.001, ^b^p < 0.01, ^c^p < 0.05 and ^d^p > 0.05 vs. control; ^e^p < 0.001, ^f^p < 0.01, ^g^p < 0.05 and ^h^p > 0.05 vs. E2 administered groups. S = Stroma, GE = Glandular epithelium, LE = Luminal epithelium.

For analysis of proliferation markers, western blotting of PR, PCNA, β-catenin was performed in uterine tissue. A significant increase in the expression of all proliferative markers (p < 0.001) was observed in the β-estradiol- administered group (36d) as compared to control group. Whereas in rats receiving K1 along with β-estradiol, the decreased expression of PR, PCNA and β-catenin was found in dose-dependent manner, as compared to β-estradiol-administered group. More significant downregulation in the expression of PR, PCNA and β-catenin (p < 0.001) was detected in rats receiving the higher dose of K1 i.e, 400 μg/kg as compared to estradiol-administered group (36 d) (Fig. 7A).

### K1 or MPA treatment antagonized estrogen-induced activation of hedgehog signaling molecules in rat uterine tissue

We performed western blotting for analysis of Hh signaling molecules such as Ihh, Shh and Gli1 expression in rat uterine tissue. In consistent to *in vitro* results, we found that the expression of Ihh, Shh, Gli 1 was continuously increased in uterine tissue of β-estradiol administered group (Fig.7B). Whereas decreased expression of Shh and Gli1 alongwith the increased expression of Ihh was observed when K1 or MPA was administered in rats receiving estradiol. In β-estradiol administered group, the up-regulated expression of Shh and Gli by ~80% and ~90% respectively (p < 0.001) was observed whereas administration of K1 or MPA down regulated the Shh and Gli1 by ~45% and ~50% respectively as compared to control group receiving vehicle alone (Fig. 7B). Surprisingly, we observed that administration of β-estradiol for 21 days effectively induced the expression of Ihh while exposure for 36 days reduced its expression.

Next, the Gli1 and PCNA expression as analyzed be by immunohistochemistry, was found to be upregulated in all endometrial compartments (stromal, luminal or glandular epithelium) in β-estradiol-administered group, as compared to vehicle- treated group. Although both K1 and MPA down regulated the expression of Gli1 and PCNA, K1 was more efficient than MPA in doing so. Rat uterine sections from K1 treated rats showed Gli1 expression to be significantly reduced in glandular epithelium by ~40% (p < 0.01) and in luminal epithelium by ~60% (p < 0.001) as compared to β-estradiol (36 d) administered group (Fig. 7C). Accordingly, these experimental observations revealed that dysregulation of Hh signaling molecules is involved in estrogen-induced rat uterine mass growth.

## Discussion

It is now understood that the Hh pathway plays an important role in all aspects of tumor progression such as development, invasion, and metastasis^40^. Alterations in Hh signaling molecules expression has also been reported earlier in hyperplasic and carcinomatous endometrium as studied by immunohistochemistry or reverse transcription–polymerase chain reaction^24^. The expression of major Hh signaling mediators, including both activators and suppressors is known to be positively correlated with ER/PR expression in normal endometrium^24^. However, the steroid- regulatory mechanisms of Hh signaling pathway as well as its physiological and pathological role, in endometrial hyperplasia are still not known.

We established a human primary atypical endometrial hyperplasial and normal endometrial cell culture to explore the Hh signaling and its regulatory mechanism. Significant alteration was observed in the expression pattern of Hh signaling molecules, including induced expression of Shh, Gli1 and the reduced expression of Ihh in EH cells as compared to NE cells. The expression of the Shh, Ihh and Gli1proteins was found to be upregulated in EH cells exposed to estrogen. Constitutive expression of pre-dominant Hh signaling ligands particularly Shh and Ihh incubated with E2 indicated existence of a potential Hh/Gli signaling cascade in EH cells. These effects were reversed by the anti-estrogenic agent (K1) and the progestin (MPA). Additionally, estrogen up-regulated the Gli1 expression as well as its nuclear translocation while K1 or MPA counteracted its expression in EH cells. Our observations suggested that K1 and MPA which act as anti-proliferative agents in endometrial hyperplasia, were involved in attenuation of the expression of Hh signaling molecules Shh and Gli1. We also demonstrated that cyclopamine (Hh inhibitor) caused growth inhibition of cultured EH cells and Gli1 silencing caused the reduced expression of proliferative markers in these cells. Similar attenuation of Hh signaling molecules in *in vivo* hyperplasia model of rat was observed under the effect of K1 or MPA. Taken together, these data suggest that Shh and Gli1 play crucial role in estrogen-induced cellular proliferation and indicate the significant involvement of canonical Hh/Gli1 pathway in endometrial hyperplasia.

Indian hedgehog (Ihh) acts as a progesterone-responsive factor, is acutely upregulated by progesterone (P4) and serves as a major mediator of progesterone signaling in the mouse uterus^41, 42^. In the endometrium of normally cycling women, the increased expression of Ihh mRNA was found during the secretory phase which suggests the progesterone -mediated regulation of Ihh^43^. There are reports that patients with endometriosis showed significantly less Ihh staining in all endometrial compartments in luteal phase of the menstrual cycle as compared to healthy volunteers, underscoring that production of Ihh is regulated by progesterone^44^. The reduced expression of Ihh as observed in EH cells in our study, can be explained by the fact that Ihh is ‘progesterone regulated factor’ and endometrial hyperplasia results from continuous estrogen stimulation unopposed by progesterone. Surprisingly, we observed increased expression of Ihh protein in EH cells in presence of estrogen as well as in presence of progesterone, although the magnitude of induction was much higher (~170%) in progesterone-treated cells. In *in vivo* study, E2 administered group (21 d) showed increased expression of Ihh similar to that in cultured human EH cells. Conversely, the continuous exposure of E2 (for longer period i.e., 36 days) or MPA along with E2 administered group did not show any significant change in Ihh expression in uterine tissue of rats. Previous studies have also reported the upregulated expression of Ihh mRNA 6 h after E2 treatment or 3 h after P4 treatment, but down-regulation was observed after longer hormonal exposure (12–48 h) in RL95–2 cells^45^. The ambiguous pattern of Ihh under hormonal treatments in our study as well as in other group’s study, indicate the need for further investigation of complex steroidal regulation of Ihh in pathological conditions of endometrium including hyperplasia.

Several lines of evidences show that Gsk3β acts as bi-potential regulator of Gli1^46^. In drosophila, it served as a negative regulator of Gli1 by phosphorylating it and promoting its degradation thereby suppressing Hh signaling^32^. However, in mammalian cells, it acts as a positive regulator of Gli1 and promotes its activation, hence, stimulates Hh signaling^33^. The regulatory mechanism of Gsk3β still remains complex and contentious because of its ambiguous role as tumor promoter or tumor suppressor^46^. There are some reports in case of carcinomas, where the suppression of Gsk3β activity induces cancer progression by stabilizing components of the β-catenin complex^46^. Inhibition of Gsk3β induces epithelial cell proliferation in xenografted human endometrium and the attenuation of Gsk3β expression in the absence of E2 was sufficient to cause a proliferative response in human endometrium implanted under the kidney capsule of nude mice^47^. It has been reported that decreased expression of Gsk3β via lithium treatment encourages estradiol-induced proliferative and morphogenic changes in the uterus of mice leading to hyperplasia^34^. Estrogen regulates the Gsk3β activity in the adult rat hippocampus via increasing the level of phosphorylation in serine of Gsk3-α and -β^48^. In addition, it has been reported that estrogen inhibits Gsk3β activity via activating a kinase downstream PI3K which in turn activates AKT, ultimately leading to regulation of Gsk3β activity by inhibitory phosphorylation^49^. We herein, observed the downregulation of Gsk3β expression in presence of estrogen, whereas K1 or MPA induced the expression Gsk3β, in hyperplasial cells, suggesting that failure of Gsk3β-mediated regulation might be a possible cause for the constitutive activation of estrogen-mediated hedgehog signaling leading to continuous proliferation in EH cells. Notably, Gsk3β activator (LY-294002) caused a decrease in Gli1 expression similar to that observed in cyclopamine -treated EH cells, thus confirming the Gli1 to be a downstream target of Gsk3β. The confocal microscopy studies indicated that Gsk3β knock down encouraged the nuclear translocation of Gli1 as like β-estradiol- treated cells while Gsk3β activator (LY-294002) reduced Gli1 expression similar to that caused by K1 or MPA. Hence, Gsk3β induction promotes inhibition of Gli1 activation (nuclear translocation) and silencing of Gsk3β significantly induced the expression of Gli1. Therefore, a decrease in the activity of Gsk3β might lead to an increased Gli1 content in the EH cells which may be responsible for enhanced proliferation of hyperplasial cells. These results demonstrated that Gsk3β directly affects the Gli1 and acts as a negative kinase regulator of Gli1 in EH cells.

**Figure 8:**
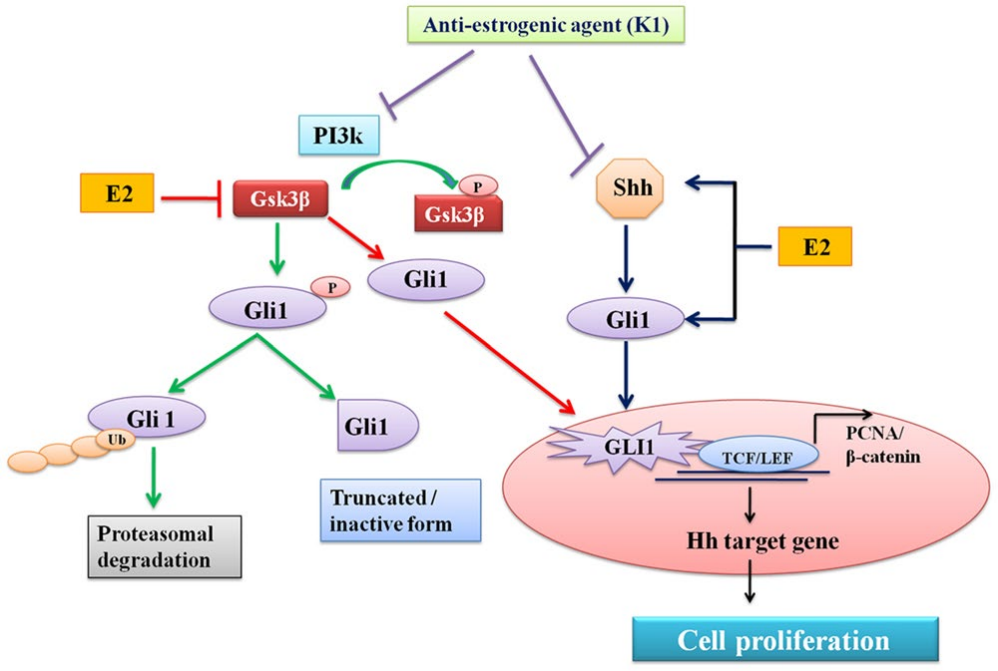
Schematic representation of functional involvement of Hh signaling molecules in estrogen- mediated endometrial hyperplasia. Exposure of estrogen induces activation of Gli1 via canonical pathway. Estrogen attenuates the Gsk3β protein expression leading to activation of Gli1which induces various target genes involved in cellular proliferation of estrogen-mediated hyperplasia progression. Anti-estrogenic agent (K1) counteracts Gli1expression via both canonical (ligand dependent) such as inhibition of Shh and non- canonical pathway (ligand independent) as activation of Gsk3β. Activation of Gsk3β might promote Gli1 phosphorylation i.e., its inactivation or proteasomal degradation^32, 57, 58^.

In conclusion, studies revealed that estrogen induces the Shh expression and promotes the activation of Gli1 in EH cells which is considered to be one of the indicators of Hh pathway activation. Additionally, it was evident that estrogen -mediated decreased Gsk3β expression encouraged the activation of Gli1. Hence, it can be inferred that estrogen affects the proliferation of endometrium via induced expression of Shh or inhibition of Gsk3β that ultimately provokes the nuclear accumulation of Gli1. Moreover, Gsk3β activator (LY-294002) caused a decrease in Gli1 expression similar to that observed in cyclopamine-treated EH cells and functional blockage of Gsk3β stimulated Gli1 expression as well as nuclear translocation. Nuclear accumulation of Gli1 clearly pointed out the involvement of canonical and/or non-canonical Hh pathway in estrogen- mediated endometrial hyperplasial cell proliferation. This was supported by the observed effect of anti-estrogenic agent (K1) and progestin (MPA) which showed growth suppressive effect through inhibition of Shh and Gli 1 along with the induction of Gsk3β expression/activation. Our study suggests that altered expression of Hh signaling-related molecules might be directly involved in the pathogenesis of endometrial hyperplasia (Fig. 8). Nevertheless, further efforts will be required with careful interpretation and analysis to determine the potential determinants and predictors of efficacy of Hh signaling molecules and Gsk3β in endometrial hyperplasia in large number of human samples. Hedgehog signaling pathway may prove as one of the vital signaling pathways playing pivotal role in controlling endometrial hyperplasic condition, making this pathway an attractive and alternative potential target for drug development.

## Materials and Methods

### Reagents and Antibodies

Modified Eagle’s medium (MEM) and [(3-(4,5-dimethylthiazol-2-yl)-2,5-diphenyltetrazolium bromide)] (MTT), Bradford, Anti-rabbit and anti-mouse cy-3 conjugated secondary antibodies, propidium iodide (PI), Annexin V-FITC (fluorescein isothiocyanate)-labeled apoptosis detection kit, β-estradiol, RIPA Buffer were purchased from Sigma-Aldrich, USA. Lipofectamine Plus reagent and Fetal bovine serum (FBS) were purchased from Invitrogen (Carlsbad, CA). Immuno-Blot™ PVDF membrane was obtained from Millipore, MA, USA. Cyclopamine, LY-294002 were purchased Calbiochem, San Diego, CA. ECL reagent was purchased from GE Healthcare, USA. Primary antibodies such as cytokeratin-7(sc-70936, anti-mouse), ERα (sc-543), PR (sc-539, anti-rabbit), PCNA(sc-56, anti-mouse), Ihh (sc-13088, anti-rabbit), Shh(sc-9024, anti-rabbit), Gli1(sc-20687, anti-rabbit), p-Gsk3β^ser9^ (sc-11757, anti-goat), Gsk3β(sc-71186, anti-mouse), β-catenin(sc-7693, anti-mouse), CyclinD1(sc-246, anti-mouse), β-actin(sc-1616,anti-goat), and secondary antibodies as horseradish peroxidase (HRP) labeled secondary antibodies, Fluorescein isothiocyanate (FITC)-conjugated secondary antibodies (anti-mouse), ABC vectastain Kit were procured from Santa Cruz (Santa Cruz, CA). All other reagents, were of the highest grade and commercially available.

Progestin medroxy progesterone acetate (MPA) tablets; DEVIRY-10mg were procured from Elder pharmaceuticals, India). MPA was procured from Sigma-Aldrich.

Anti-estrogenic agent K1 (2-(piperidinoethoxyphenyl)-3-(4-hydroxyphenyl)-2H-benzo (b) pyran) was synthesized by Medicinal Chemists of the CSIR-CDRI, Lucknow^50, 51^.

### Endometrial tissue collection

Endometrial hyperplasial and normal endometrium sample were collected in the operating room of the Department of Obstetrics and Gynecology, King George’s Medical University, Lucknow, India. A specific informed consent was obtained from the patient, and the study was approved by the Ethics Committee of King George’s Medical University, Lucknow India. We obtained endometrial hyperplasial tissue (n = 7) from the patient who had endometrial hyperplasia with abnormal uterine bleeding and was undergoing total abdominal hysterectomy. Normal endometrial sample (n = 6) was collected from the patients undergoing hysterectomy for uterine prolapse reasons. All experiments on human subjects were performed in accordance with relevant guidelines and regulations.

Histopathological examination such as gland-to-stroma ratio, gland’s size and shape, cytologic atypia, nuclear/cytoplasmic ratio, hyperchromatosis by simple staining and expression of ER, progesterone receptor (PR) and proliferation marker (Ki67) by immune-histochemistry were carried out by Gynecologists and Pathologists from Department of Obstetrics and Gynecology and Department of Pathology, King George’s Medical University, who confirmed the category of samples as hyperplasia to be of atypical type.

### Primary culture of endometrial cells

Cell isolation was based on the methods as described by Genc *et al*.^52^ with slight modification^52^. Tissues were collected in MEM, minced into 1 mm pieces and incubated with 1 mg/ml collagenase and DNase (2 mg/ml) in MEM for 2 h at 37 °C in CO_2_ incubator with regular mixing. Digested tissue was mechanically dissociated through a 1 ml tip and re-suspended in 2 ml of fresh MEM. Cells were separated from tissue clumps and debris as described earlier^53^.

The isolated cells were highly viable. These cells in primary culture showed a typical epithelial morphology with highly packed polygonal cells. To confirm the epithelial characteristics of primary hyperplasial cells, expression of an epithelial marker (cytokeratin-7) was checked by immunofluorescence (Supplementary Fig. 1D).

### Cell viability assay

Analysis of cell viability was done by MTT assay. Cells were seeded (3 × 10^3^ cells/well) into 96- well plate MEM containing 10% FBS, 5% CO_2_ and treated with different concentration of anti-estrogenic agent K1 (1 μM, 2.5 μM, 5 μM, 7.5 μM, and 10 μM) and cyclopamine (1μM-50μM, and100μM) for 48 h. The experiments were performed three times with five replicates in each. The MTT assay was performed as described earlier^53^.

### Western Blotting

Cells were plated in T-25 cm^2^ flasks and made quiescent at confluence by incubation in serum free MEM and stripped FBS for 24 h. Control and treated cells of different groups were lysed in RIPA buffer and supernatant was collected by centrifugation (15,000 rpm for 15 minutes).

For *in vivo* experiments, the frozen endometrial hyperplasial tissue of control vehicle treated and different treated group were thawed, washed with PBS and homogenized in RIPA buffer for approximately 5 min under ice-cold conditions and incubated overnight in −20 °C and supernatant was collected by centrifugation.

Supernatant containing soluble protein was quantified by Bradford Reagent^54^ and processed as described earlier^55^. Experiments were performed on three independent patient’s samples and were repeated three times for each patient sample. Quantitation of band intensity was performed by densitometry using Quantity One H software (v.4.5.1) and a Gel Doc imaging system (Bio-Rad).

### Immunofluorescence imaging by fluorescence microscopy

Primary human EH cells were grown on coverslips in 12-well plate. Cells were treated with vehicle, E2 (10 nM), cyclopamine (20 μM) or cyclopamine +E2 for 24 h to check the expression of proliferative markers. Cells were processed as described earlier^53^. Images were captured at 20× using Leica fluorescence microscope.

Further, to check the Gsk3β expression, EH cells were treated with vehicle, E2 (10 nM), K1 (5 μM) or MPA (5 μM) for 24 h and cells were processed for immunofluorescence as previously method. Images were grapsed at 40× using Nikon fluorescence microscope and fluorescence intensity was quantified by NIS-Elements software.

In addition, co-expression of Gsk3β and Gli1 was demonstrated in EH cells transfected with Gsk3β siRNA or scrambled siRNA or E2- treated or LY-294002- treated cells. Images were captured at 20× using Leica fluorescence microscope. Image fluorescence intensity was measured by Leica LAS Image analysis software. All experiments were repeated at least three times.

### Confocal microscopy

To detect sub-cellular localization of Gli1 in EH cells, cells were seeded on coverslips in 12-well plate and treated with vehicle, E2 (10 nM), 5 μM of K1 and MPA (5 μM) for 48 h. Cells were then fixed in 4% p-formaldehyde and permeabilized with 0.1% Triton X-100. Cells were washed with PBS and blocked with 1% BSA and incubated with Gli1 antibody for overnight followed by 1 h incubation with fluorescence-tagged secondary antibody (Cy-3), then mounted on slides with SlowFade® Gold Antifade reagent with DAPI (molecular probes, life technologies). Images were captured at 63X using Carl Zeiss LSM 510 META microscope^53^. Cells not exposed to primary antibodies served as negative controls. Fluorescence intensity of images was quantified by LSM Image Browser software. Similar procedure we followed to detect the nuclear translocation of Gli1 in presence of LY-294002, E2 and Gsk3β- transfected EH cells.

### Annexin-V/propidium iodide labeling and flow cytometry assay for apoptosis

EH Cells (2 × 10^5^ cells per ml) were cultured in 6-well plates and incubated with cyclopamine for 24 h and processed as described earlier^53^. Adherent and non-adherent cells were probed with FITC-conjugated Annexin-V and PI for 10 min. The staining profiles were determined with FACScan and Cell-Quest software. The experiments were performed three times.

### siRNA transfection

For siRNA transfection, primary human EH cells were seeded in T-25 cm^2^ culture flasks and allowed to attain confluency of 70–80%. Cells were then incubated in antibiotic-free medium overnight, followed by transfection with siRNA duplex–Lipofectamine according to manufacturer’s protocol. Briefly, 50 pmols of Gli1 siRNA or Gsk3β siRNA were diluted in 100 μl of transfection medium and added with 100 μl of diluted Lipofectamine® RNAiMAX Reagent. The mixture was allowed to incubate at room temperature for 30 min^55^. Cells were then transfected with scram siRNA or Gli1 siRNA or Gsk3β siRNA for 6 h at 37 °C in a CO_2_ incubator followed by a different treatment for 48 h. Transfection efficiency of siRNA was also determined by western blotting.

### Animal preparation and treatment schedule

Young adult rats (Sprague Dawley strain) of body weight of 150 g were used in this study. Animals were housed under uniform animal husbandry conditions with free access to pelleted food and water. All animal procedures were carried out as per the guidelines provided by the Institutional Animal Ethics, Use and Care Committee. Prior approval was obtained from the Institutional Animal Ethics Committee (IAEC) of Central Drug Research Institute, Lucknow, India for animal experimentation.

Rats were ovariectomized bilaterally under ether anesthesia and given a rest period of 2 weeks. Following the rest period, rats were divided into various groups (7 rats per group): group I received olive oil and gumacacia as vehicle, group II received β-estradiol (20 μg/kg body weight, in olive oil, subcutaneously) for 21 days, groups III received β-estradiol (20 μg/kg body weight, in olive oil, subcutaneously) for 36 days and groups IV, V, VI received, in addition to estradiol, K1 at 100 μg/kg, 200 μg/kg 400 μg/kg body weight doses (in gum acacia, orally) was given after 21 days of treatment schedule, and groups VII received, in addition to estradiol, MPA 25 mg/kg body weight (in water, dose-orally) after 21 days of treatment schedule^37^. All treatments were given for 14 days. Animals were euthanized 24 h after the last treatment. Uteri were collected, weighed, and stored at −80 °C until analysis. A mid portion of a single horn of each uteri was preserved in 4% paraformaldehyde for histological and histomorphometric analysis.

### Immunohistochemistry

Immunohistochemical analysis was performed as described earlier^56^. Briefly, tissue fragments were fixed in 10% formaldehyde/PBS and embedded in paraffin. Tissues were cut into 5 µm sections and mounted on glass slides and processed with minor modifications as afterprimary antibody incubation and washing, the slides were incubated with biotinylated anti-rabbit IgG (ABC vectastain Kit; Vector Laboratories, Burlingame, CA, USA; 1:200 in PBS) and then treated with avidin-peroxidase (ABC vectastain Kit) and revealed with diaminobenzidine (ABC vectastain Kit), according to the manufacturer’s instructions. Tissues were counterstained with hematoxylin, mounted with DPX (Sigma-Aldrich) and visualized through a Nikon Eclipse-800 microscope. The staining intensity of all these proteins in glandular epithelium, luminal epithelium and stromal compartment were quantified by image analysis software Image-Pro Plus 4.0 (Maryland, USA) and results were expressed as % image analysis score.

### Statistical analysis

Results are expressed as Mean ± SEM for at least three separate determinations for each experiment. Statistical significance was determined by ANOVA and Newmann Keul’s test. p values less than 0.05 were considered significant.

## Electronic supplementary material

Supplementary Information
