# Supplementary material for: The regulation of Hh/Gli1 signaling cascade involves Gsk3β- mediated mechanism in estrogen-derived endometrial hyperplasia
Source: Sci Rep. 2017 Jul 26;7:6557. doi: 10.1038/s41598-017-06370-1 (PMC5529438; doi:10.1038/s41598-017-06370-1)

The regulation of Hh/Gli1 signaling cascade involves Gsk3β- mediated mechanism in estrogen-derived endometrial hyperplasia

Jyoti Bala Kaushal[1,4], Pushplata Sankhwar[2] , Suparna Kumari[1], Pooja Popli[1], Vinay Shukla[1,4] , Mohd. Kamil Hussain[3], Kanchan Hajela[3, 4], Anila Dwivedi[1, 4*]

[1]Division of Endocrinology, CSIR-Central Drug Research Institute, Lucknow-226031, U.P., India

[2]Department of Obstetrics & Gynecology, King George's Medical University, Lucknow-226001, U.P., India

[3]Division of Medicinal & Process chemistry, CSIR-Central Drug Research Institute, Lucknow-226031, U.P., India

[4]Academy of Scientific and Innovative Research (AcSIR), New Delhi-110025, India.

*Corresponding Author: Anila Dwivedi, Senior Principal Scientist and Head, Female Reproductive Biology Laboratory, Endocrinology Division, CSIR-Central Drug Research Institute, Lucknow-226031, , UP, India.

Tel: +91 0522 277 2486; Fax: +91 0522 2623405/2623938, E-mail: anila_dwivedi@cdri.res.in

Supplementary Information

Supplementary Figure 1

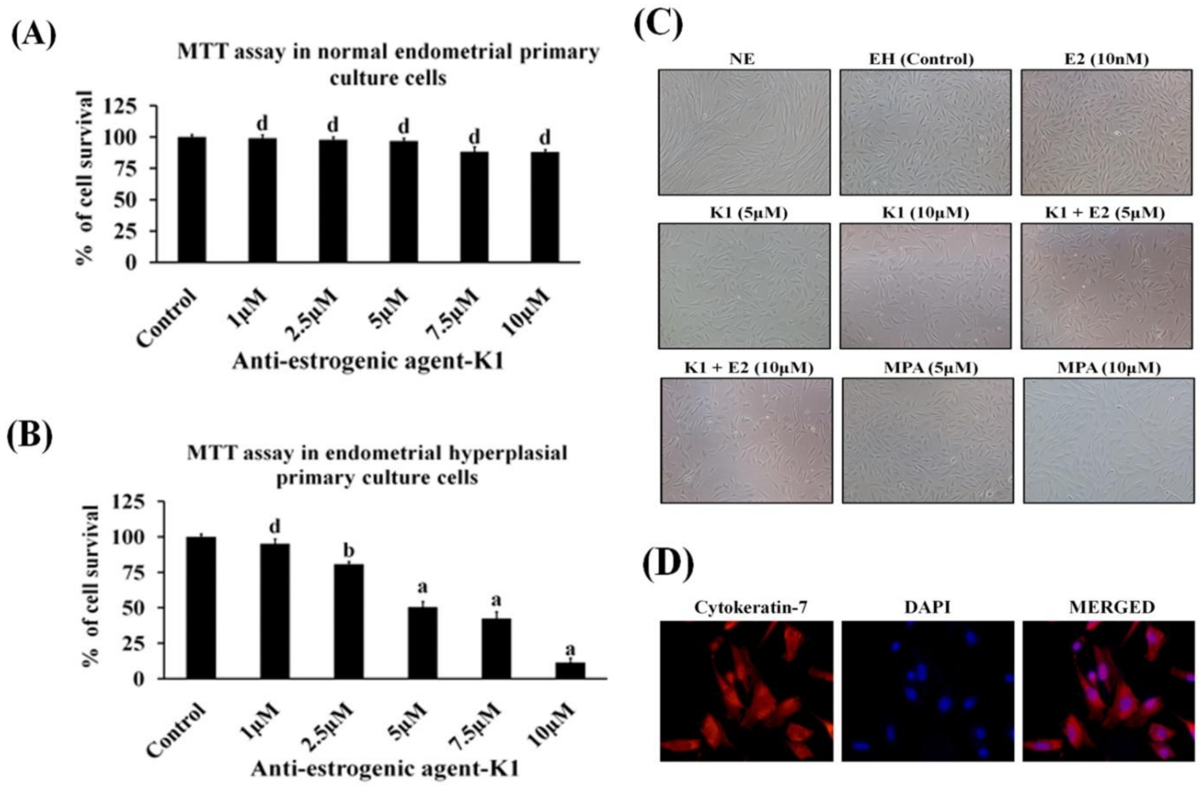

Supplementary Figure 1. Anti-estrogenic agent (K1) mediated growth suppression in primary human endometrial hyperplasial cells. Cellular growth pattern of primary human **(A)** normal endometrial cells (NE) and **(B)** endometrial hyperplasial (EH) cells. Cells were treated with varying doses of K1 (2-[piperidinoethoxyphenyl]-3-[4-hydroxyphenyl]- 2H-benzo(b) pyran), i.e., 1.0, 2.5, 5.0, 7.5 and 10μM for 48 h. The level of cell proliferation was measured using MTT assay. The percentage of viable cells was calculated as the ratio of treated cells to the control cells. Results are expressed as mean ± SEM, n = 5. p values are a-$p<0.001$, b-$p<0.01$, c-$p< 0.05$ and d-$p>.05$ vs. Control. **(C)** Representative images of proliferation and morphological changes in NE cells and EH cells treated with vehicle, E2 (10nm) , K1 (5μM or 10μM) in the absence or in presence of E2 (10nM) and MPA (5μM or 10μM) for 48 h in MEM containing 10% charcoal stripped FBS. **(D)** The primary human EH cells were characterized by the

expression of cytokeratin-7, an epithelial marker, by immunofluorescence. EH cells were fixed, permeabilized, incubated with cytoketratin-7 antibody for overnight, as described experimental procedure section and cell images were grasped using a Nikon fluorescence microscope at 40X.

Supplementary Figure 2

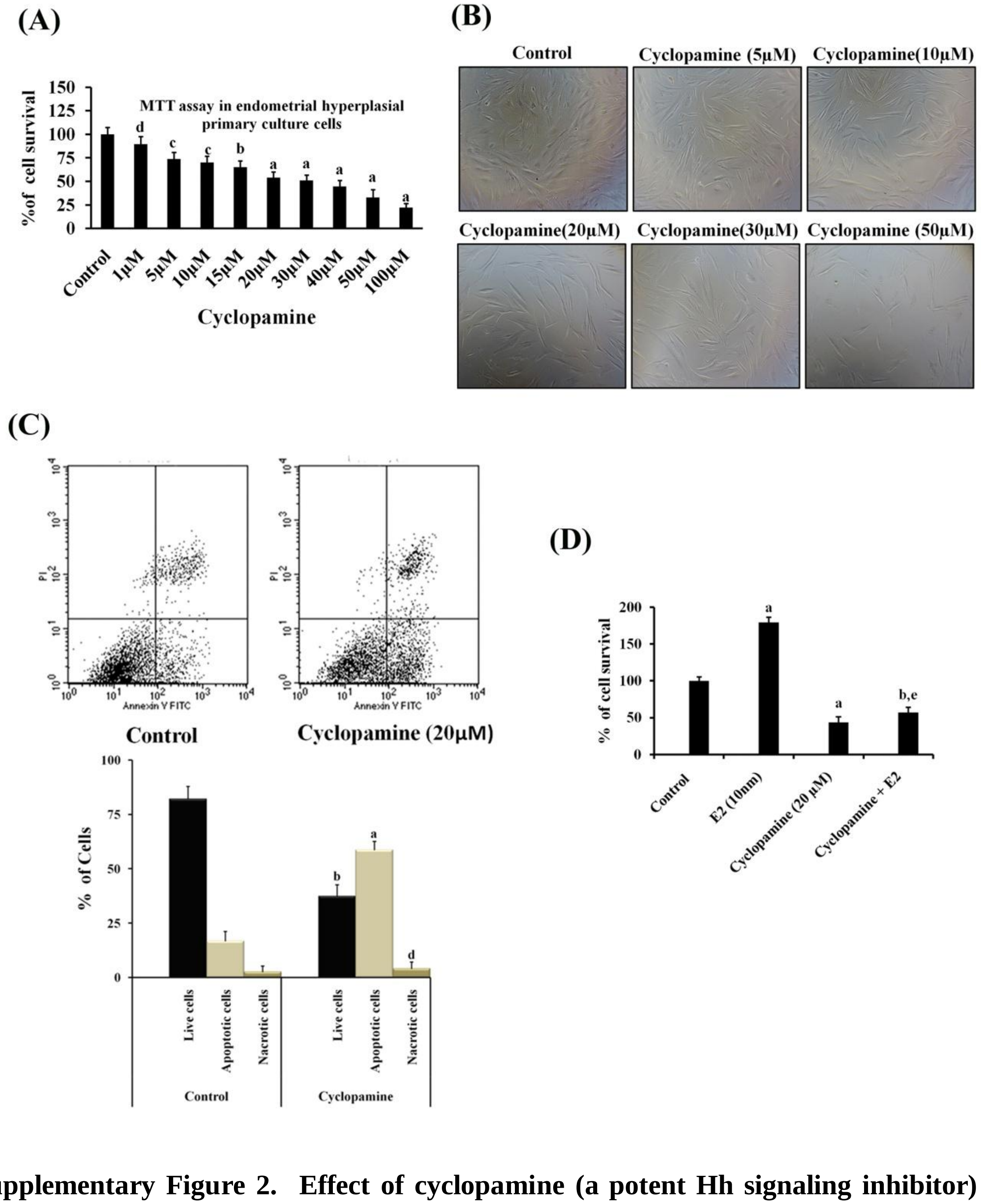

Supplementary Figure 2. Effect of cyclopamine (a potent Hh signaling inhibitor) on cell survival of primary human endometrial hyperplasial cells. (A) EH cells were treated with

various concentrations of cyclopamine (1.0, 10, 20, 30, 40, 50µM, and 100µM) for 48h. Cell viability was measured using MTT cell viability assay. The percentage of viable cells was calculated as the ratio of treated cells to the control cells. Results are expressed as mean ± SEM, n = 5. p values are a-$p<0.001$, b-$p<0.01$, c-$p< 0.05$ and d-$p>.05$ vs. control. **(B)** Representative images of proliferation and morphological changes in primary human endometrial hyperplasial cells treated with cyclopamine (10 µM, 20µM, 30µM, 50µM) concentrations. **(C)** Analysis of apoptosis in human EH cells treated with cyclopamine by flow cytometric analysis of annexin-V/PI-stained cells after 24 h culture (AV+/PI – intact cells; AV/PI+ – nonviable/necrotic cells; AV+/PI and AV+/PI+ – apoptotic cells). Representative images are shown in the upper panel and the percentage of cell fraction was calculated based on three independent experiments. p values are a-$p<0.001$, b-$p<0.01$,and d-$p>0.05$ versus control. **(D)** Representative graph of cell survival assay showing cyclopamine mediated inhibition in E2 induced cell proliferation. Results are expressed as mean ± SEM, n = 3. p values are a-$p<0.001$, b- $p<0.01$, vs. Control or e-$p<0.001$ compared to E2.

Supplementary Figure 3.

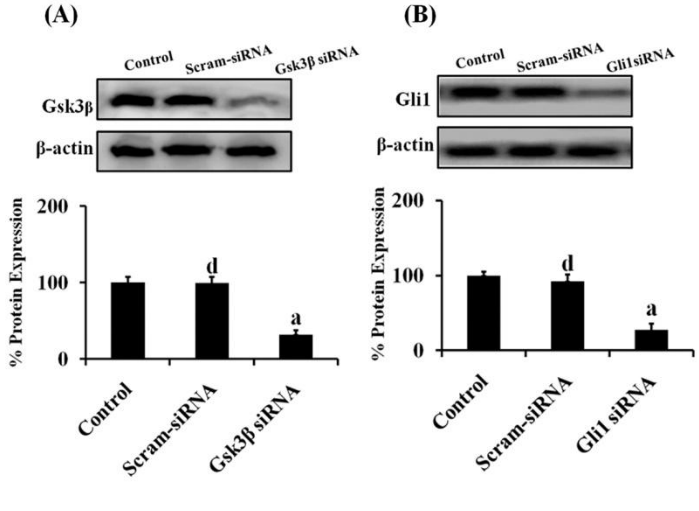

Supplementary Figure 3. Demonstration the transfection efficiency of Gsk3β or Gli1 siRNA versus Scrambled siRNA or Controls. (A) Representative western blots showing the knockdown efficiency of Gsk3β siRNA. For Gsk3β silencing, EH cells were transfected with negative control scrambled siRNA or with 50 pmols of Gsk3β siRNA and allowed to incubate for 6 h at 37°C in a CO_2 incubator and after 24 h incubation whole cell lysate were collected and

proceed for SDS-PAGE and western blotting. **(B)** Representative western blots showing the knockdown efficiency of Gli1 siRNA as described procedure above.

Supplementary Figure 4.

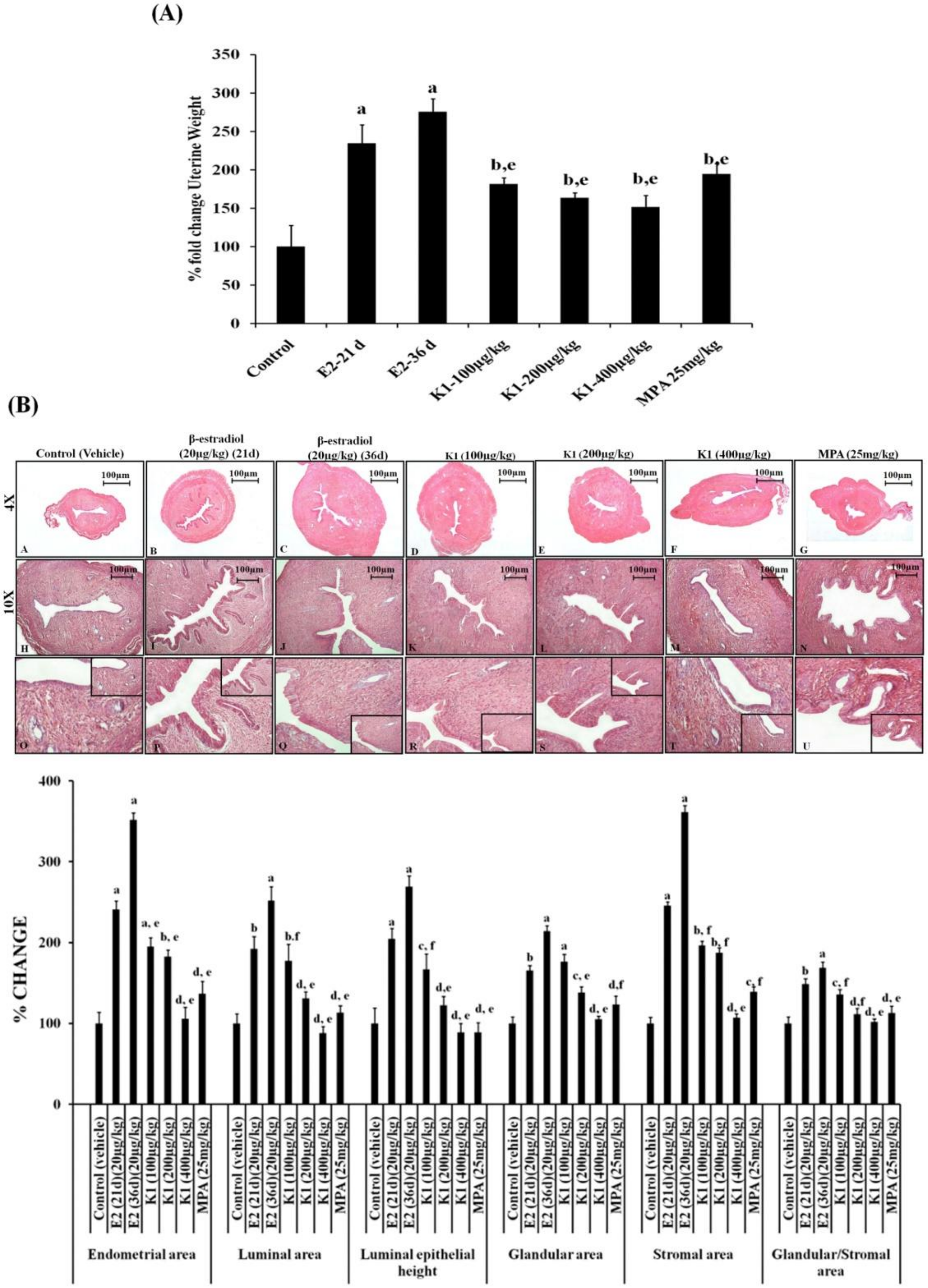

Supplementary Figure 4. Effect of anti-estrogenic agent (K1) or progestin (MPA) treatment on uterine mass growth of rat and expression profile of proliferative markers. Ovariectomized rats were treated with β-estradiol (E2) (20 g/ kg) or E2 alongwith K1 at 100 µg/kg or 200 µg/kg or 400 µg/kg or MPA 25mg/kg for 14 days. Animals were euthanized 24 h after the last treatment and uteri were collected. **(A**) Data represent the wet uterine weight (% fold change) of control (vehicle) or E2 or E2 alongwith K1 or E2 alongwith MPA administered group. Results are expressed as mean ± SEM, n=3. p values are a- p<0.001, b—p<0.01, c—p<0.05 and d—p>0.05 vs. control and e- p<0.001, f—p<0.01, g—p<0.05 and h—p>0.05 vs.E2 (36 D) group. **(B)** Cross-sectional view of rat uteri showing histological changes at ×4 (A-G) and ×10 (H-N) magnification with crop area (O-U), obtained from control (A, G and H), E2 [21d] (B, I and P), E2 [36d] (C, J and Q), E2 alongwith K1 100µg/kg (D, K and R), E2 alogwith K1 200µg/kg (E, L and S), E2 alongwith K1 400µg/kg (F, M and T) or E2 alonwith MPA 25mg/kg (G, N and U) administered rats. **(C)** Histomorphometric analysis of formalin-fixed, paraffinembedded rat uterine sections of various groups. Values are expressed as mean ± SEM (n = 6). p values are a-p<0.001, b-p<0.01, c-p<0.05 and d-p>0.05 vs. control and e- p<0.001, f-p<0.01, g-p<0.05 and h-p>0.05 vs. E2 (36d) administered groups.

Supplementary Figure 5.

Full blots for Figure 1

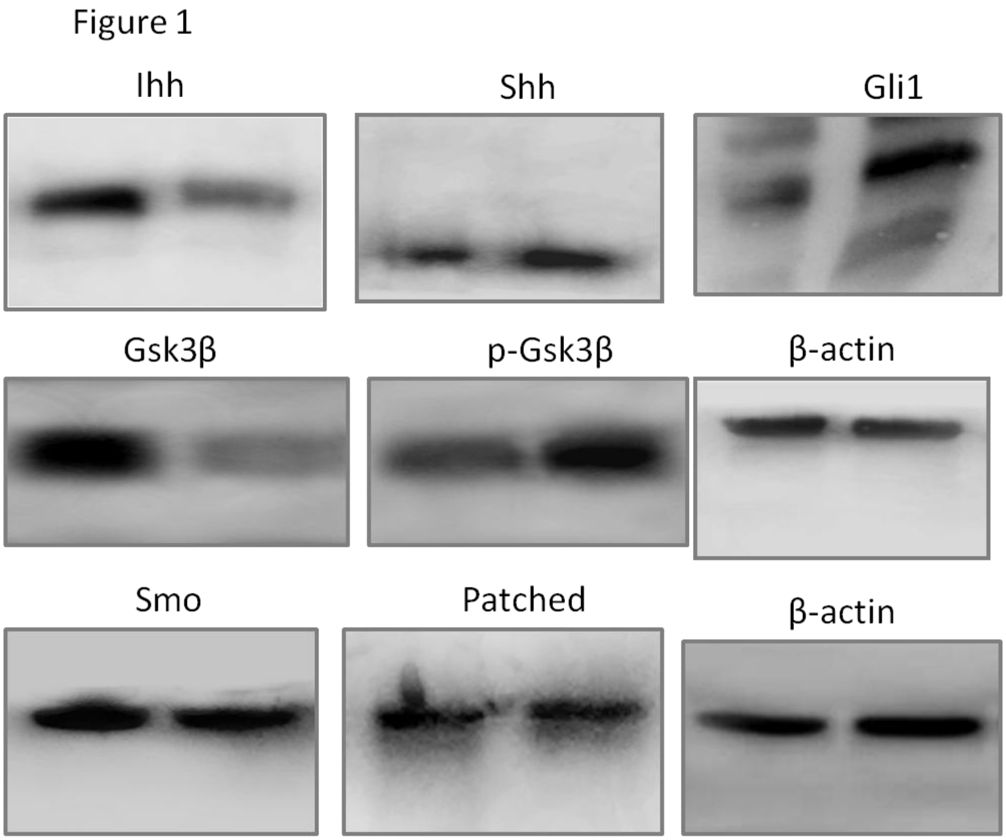

Full blots for Figure 2

Figure 2 (B)

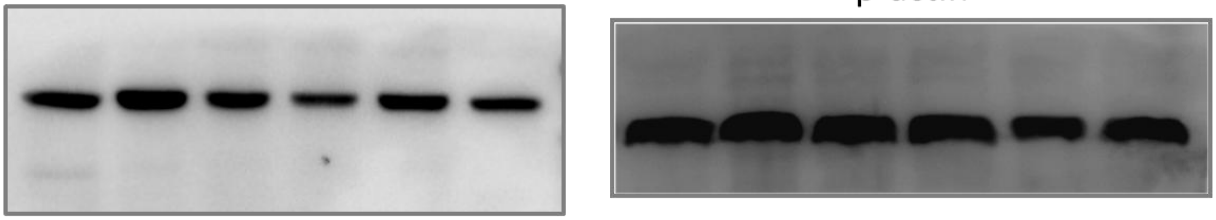

Figure 2 (D)

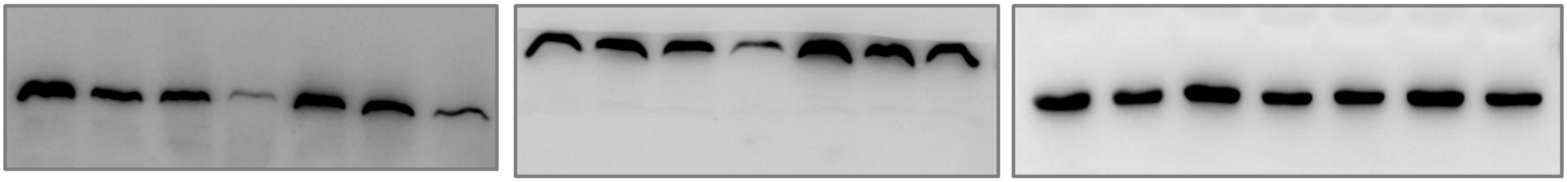

Full blots for Figure 3

Figure 3

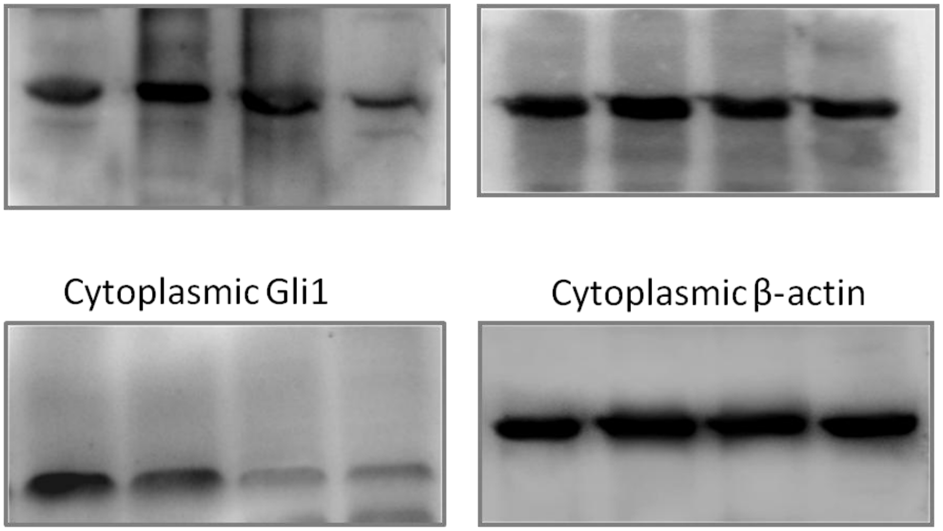

Full blots for Figure 4

Figure 4 (A)

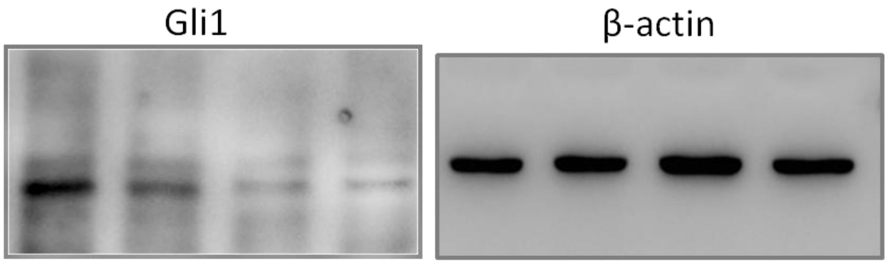

Figure 4 (B)

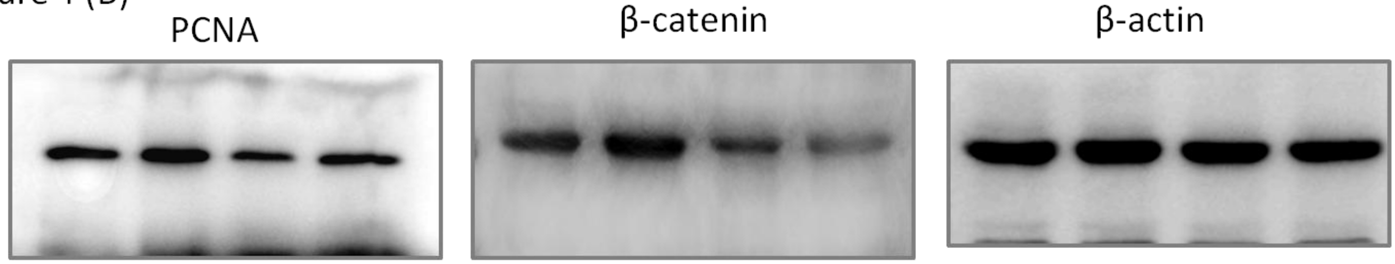

Figure 4 (D)

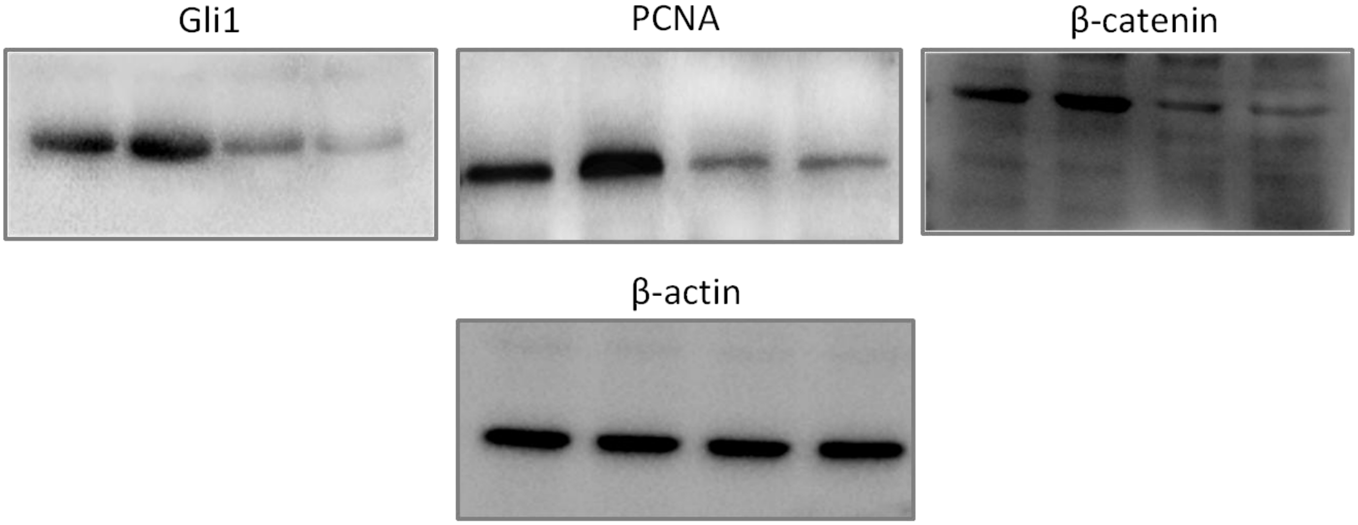

Full blots for Figure 5

Figure 5

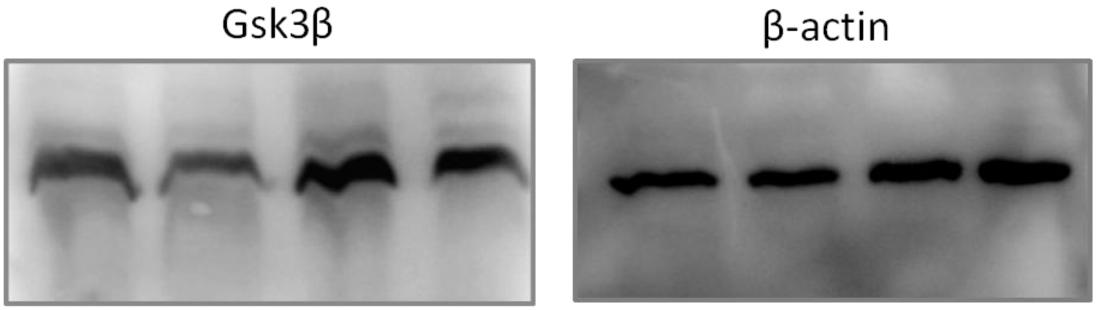

Full blots for Figure 6

Figure 6 (A)

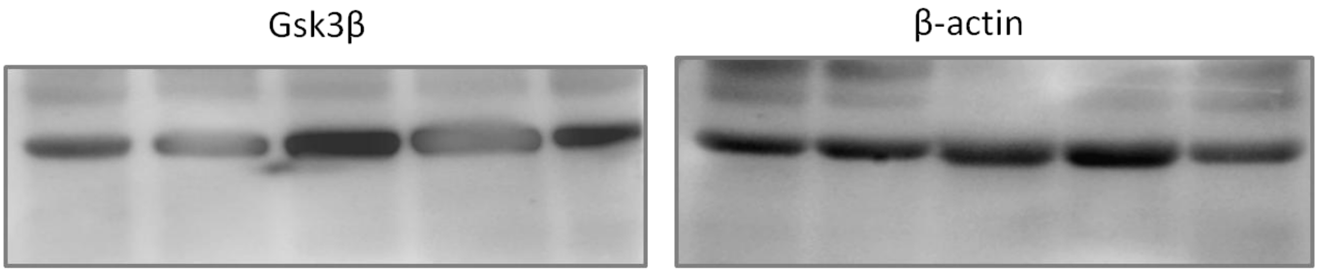

Figure 6 (B)

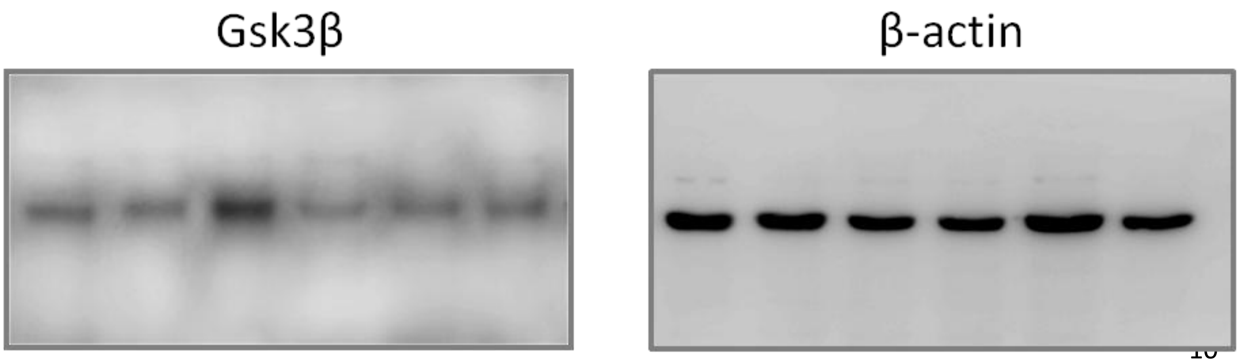

Figure 6 (E)

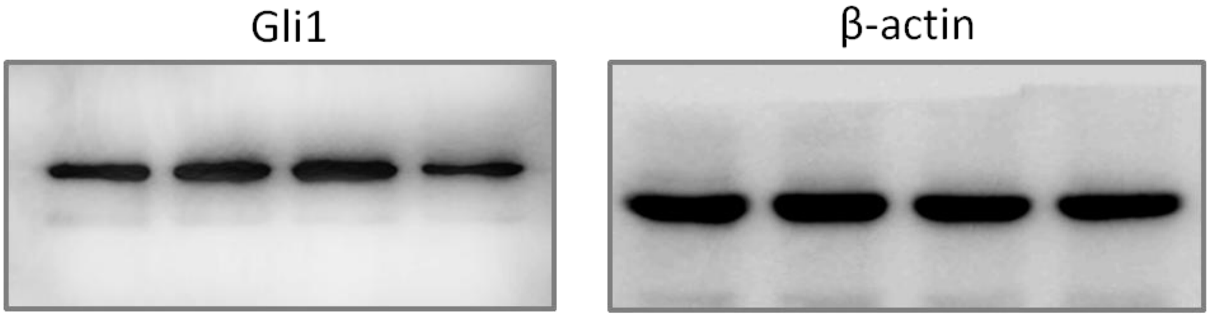

Full blots for Figure 7

Figure 7 (B)

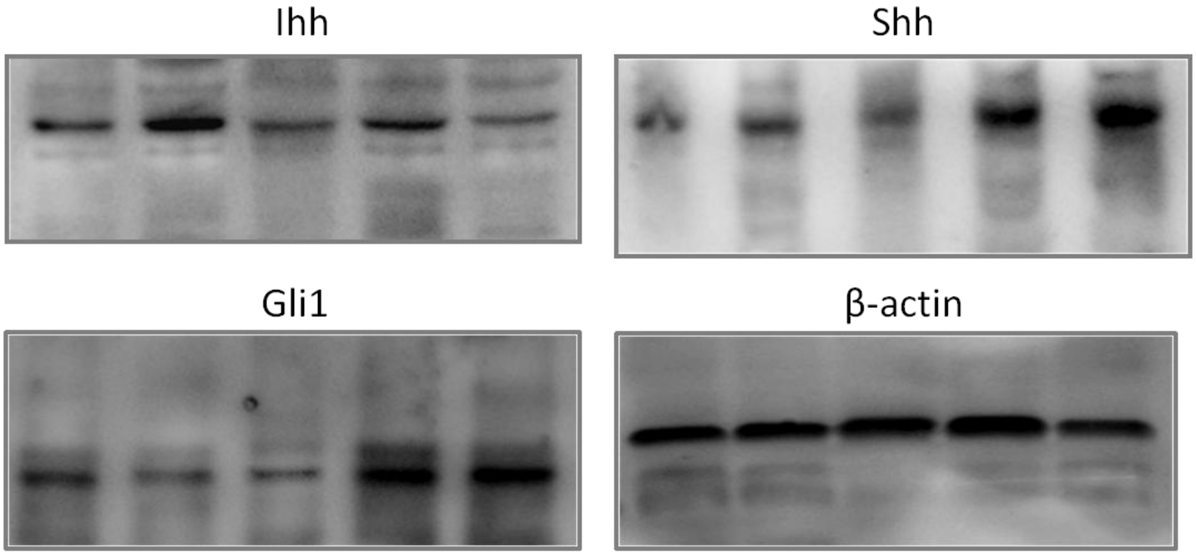

Supplement: Supplementary file 1 — Supplementary Information [file 41598_2017_6370_MOESM1_ESM.pdf]
